# Exercise, physical activity, physical fitness, and humoral responses to influenza vaccination in adults: a systematic review and meta-analysis

**DOI:** 10.3389/fimmu.2026.1950491

**Published:** 2026-10-08

**Authors:** Xiaoman Hao, Tianyuan Yu, Weibao Liang, Yue Zong, Yasong Zhang, Yu Hou, Zhijia Sun

**Affiliations:** 1 School of Physical Education, Chengdu Vocational University of the Arts, Chengdu, China; 2 School of Information Technology, Vitebsk State University named after P.M. Masherov, Vitebsk, Belarus; 3 South China Normal University, Guangzhou, China; 4 School of Physical Education, Guangdong Technology College, Zhaoqing, China; 5 Department of Basic Medicine, Langfang Health Vocational College, Langfang, China; 6 College of Sports Science, Shenyang Normal University, Shenyang, China; 7 Hebei Sport University, Shijiazhuang, China

**Keywords:** exercise, hemagglutination inhibition, humoral immunity, influenza vaccination, meta-analysis, physical activity, physical fitness, systematic review

## Abstract

**Background:**

Exercise, habitual physical activity, and physical fitness have been proposed as behavioral adjuvants that could improve influenza vaccine immunogenicity, but individual studies are small and inconsistent.

**Objective:**

To evaluate the effects of assigned acute and chronic exercise interventions, and the associations of habitual activity and fitness, with humoral responses to influenza vaccination in adults.

**Methods:**

We searched PubMed, Web of Science, Embase, Cochrane Library, Scopus, and SPORTDiscus from inception to May 1, 2026. Randomized evidence was analyzed separately for acute exercise and chronic training; nonrandomized interventions and observed activity or fitness were considered separately. Risk ratios (RRs) and mean differences (MDs) in log_2_ hemagglutination inhibition (HAI) titers were synthesized when compatible numerical data were available. Design-appropriate risk-of-bias tools and GRADE were applied.

**Results:**

Twenty-two reports were included. Seven randomized reports provided primary quantitative estimates, and five contributed to at least one meta-analysis of two or more trials. Acute exercise did not show a clear effect on H1N1 seroprotection (RR 0.88, 95% CI 0.65–1.21; k = 3) or H3N2 seroprotection (RR 1.04, 0.91–1.20; k = 3), or on H1N1 seroconversion (RR 1.30, 0.58–2.92; k = 2) or H3N2 seroconversion (RR 0.84, 0.50–1.40; k = 2). Chronic-training titer estimates were imprecise for H1N1 (MD 0.50 log_2_ units, −0.64 to 1.64; k = 2), H3N2 (0.27, −0.17 to 0.72; k = 2), and influenza B (−0.14, −0.64 to 0.36; k = 2). Other primary comparisons were supported by single trials. Certainty was low or very low.

**Conclusion:**

The available randomized evidence does not establish an immunogenicity benefit of acute exercise or chronic training, but does not demonstrate equivalence or exclude benefit. Observational associations remain vulnerable to confounding. Larger trials with complete numerical reporting are needed; these antibody findings do not establish effects on clinical influenza.

**Systematic Review Registration:**

https://www.crd.york.ac.uk/PROSPERO/view/CRD420261425732, identifier CRD420261425732.

## Introduction

Seasonal influenza causes a substantial and recurrent global burden of illness. Modeling studies estimate that influenza-associated respiratory deaths number in the hundreds of thousands each year, with the highest mortality rates in adults aged 75 years and older, and the Global Burden of Disease analyses attribute tens of millions of episodes of lower respiratory tract infection to influenza annually (1, 2). Annual vaccination remains the principal preventive strategy, but its effectiveness is only moderate and varies markedly by season, by circulating strain and by the age and health of the recipient (3).

A large part of that variability is immunological. Antibody responses to inactivated influenza vaccine decline with age: a quantitative review of trials in older adults found consistently lower seroconversion and seroprotection rates than in younger recipients (4). The underlying changes, collectively described as immunosenescence, include thymic involution, contraction of the naive T-cell repertoire, impaired germinal-center and B-cell function and a chronic low-grade inflammatory state (5–7). These changes translate directly into weaker vaccine-induced humoral immunity, and they are one reason why strategies to improve vaccine responses in older adults are a persistent public-health priority (8–10). Serum hemagglutination inhibition (HAI) antibody remains the most widely used immunological read-out in this field: a post-vaccination titer of at least 1:40 has been associated with roughly a 50% reduction in the risk of infection in the classical challenge studies, and later analyses have refined, without displacing, that relationship (11–13).

Not all of the variation in vaccine response is fixed. Host and behavioral factors, including nutritional status, psychological stress, sleep, and physical activity, may influence the immune response to vaccination (14). Chronic psychological stress is associated with poorer antibody responses to influenza vaccine (15–17). Evidence that sleep after hepatitis A vaccination supports immunological memory provides broader vaccine-related context rather than direct evidence about influenza vaccination (18). Interest in behavioral adjuvants follows naturally: interventions delivered near vaccination could potentially improve immunogenicity without reformulating the vaccine.

Exercise is the most extensively studied candidate. The view that exercise suppresses immunity has been substantially revised; contemporary syntheses argue that acute exercise transiently redeploys immune cells rather than depleting them, and that regular physical activity is associated with better immune competence across the lifespan (19–21). Habitual physical activity and higher cardiorespiratory fitness are associated with attenuated features of immunosenescence, including greater thymic output and a more favorable T-cell phenotype (22–25). Two broad mechanistic rationales have been proposed for a vaccine-specific effect. Acute exercise performed close to immunization may act as an adjuvant by increasing local blood and lymph flow, enhancing antigen delivery to draining lymph nodes, and by generating a transient neuroendocrine and inflammatory milieu, mediated in part by catecholamines and by muscle-derived interleukin-6, that favors antigen presentation and lymphocyte trafficking (26–29). Long-term training and habitual activity may instead act by improving the baseline immune environment, including a reduction in chronic low-grade inflammation (30, 31). Physical activity is already recommended for adults of all ages on other grounds, so any additional benefit for vaccine responses would strengthen an existing public-health message rather than create a new one (32–34).

The empirical picture is, however, unsettled. Individual studies examine exposures ranging from a single exercise bout near vaccination to months of training or pre-existing activity and fitness, and use different antibody metrics and follow-up schedules. An individual-participant-data meta-analysis has already examined acute exercise and habitual activity in relation to influenza vaccine responses, while a broader meta-analysis has examined exercise and vaccination across vaccine types (35, 36). A systematic review of physical activity and immune function also included vaccination outcomes, and a Cochrane review specifically evaluated exercise before influenza vaccination (37, 38). The contribution of the present review is therefore not the first synthesis of this question, but a design- and exposure-specific reassessment of strain-specific humoral outcomes, numerical extractability, risk of bias, and certainty.

We therefore asked whether assigned acute exercise or chronic exercise training changes humoral responses to influenza vaccination in adults, and whether observed habitual activity or fitness is associated with those responses. Acute exercise, chronic training, observed activity or fitness, and exercise-based mind-body interventions were distinguished rather than treated as interchangeable exposures. Older adults were identified as a subgroup of interest.

## Methods

### Protocol, registration and reporting

The review protocol was registered in PROSPERO (CRD420261425732). Reporting follows the PRISMA 2020 statement and the PRISMA-S search-reporting extension (39, 40). Data handling and synthesis were informed by the Cochrane Handbook for Systematic Reviews of Interventions (41). During revision, the analyses were separated by exposure duration and study design, ambiguous source values were removed from primary estimates, and the certainty assessments were reconsidered. These revisions and the additional sensitivity analyses are reported as amendments, not as prespecified analyses.

### Eligibility criteria

Studies were eligible if they enrolled adults aged 18 years or older who received influenza vaccination. Eligible exposures were acute exercise, long-term exercise training, habitual physical activity, physical fitness, and Taiji, Qigong or other exercise-based interventions in which the exercise contrast could be separated from co-interventions. Eligible comparators were rest or no exercise, lower activity or lower fitness, non-trained participants, waitlist or usual lifestyle, or an eligible active control condition. Continuous exposure-antibody associations were eligible, and studies reporting three or more ordered exposure levels were retained for exposure-gradient analysis.

Primary humoral outcomes were HAI antibody titers, geometric mean titers (GMTs), fold rise, seroprotection (postvaccination HAI titer ≥1:40), and seroconversion (a ≥4-fold rise). Secondary humoral outcomes included influenza-specific IgG, IgM, IgA, and neutralizing antibody. Cell-mediated immunity and vaccine reactions were supplementary outcomes and did not substitute for a humoral outcome when determining review eligibility. Randomized trials, nonrandomized exercise interventions, and observational exposure studies with an eligible comparison were considered.

We excluded non-adult samples without separable adult results, vaccines other than influenza vaccine, multicomponent interventions without an identifiable exercise contrast, studies without an eligible exposure comparison or humoral response, animal studies, reviews, and duplicate outcome reports. Availability of compatible numerical data determined inclusion in a quantitative synthesis rather than automatically determining eligibility for narrative review. Reports from overlapping cohorts were linked, and a cohort was not entered twice in the same synthesis. Report counts and participant counts were distinguished; a sum across overlapping reports was not interpreted as the number of unique participants.

### Information sources and search strategy

Six electronic databases were searched from inception to May 1, 2026: PubMed (MEDLINE), Web of Science Core Collection, Embase, the Cochrane Library (CENTRAL), Scopus, and SPORTDiscus (EBSCOhost). Each strategy combined exercise/activity/fitness, influenza vaccination, and humoral immune-response concepts. No language or date restrictions were applied at the search stage. The full database-specific strategies are reproduced in **Supplementary Table S1**. The search was restricted to these bibliographic databases; trial registries and gray-literature sources were not searched. The humoral-outcome block may have reduced sensitivity when eligible outcomes were not described in searchable fields, and using several databases does not remove that shared restriction.

**Supplementary Table S2** provides the numerical estimates and analysis status, **Supplementary Table S3** supports the design-specific risk-of-bias judgments, **Supplementary Table S4** reports sensitivity analyses, **Supplementary Table S5** describes nonpooled findings and observational associations, and **Supplementary Table S6** provides the GRADE evidence profiles. **Supplementary Table S7** documents numerical provenance, reconstruction methods, and exclusions, including the exclusion of exploratory figure-derived readings from Hallam et al.

### Study selection and data collection

Records were managed and deduplicated in Zotero 9.0. Two reviewers (Xiaoman Hao and Weibao Liang) independently screened titles and abstracts and assessed potentially eligible full texts against the eligibility criteria. The same reviewers independently extracted data and assessed risk of bias. Disagreements were resolved through discussion and, when necessary, adjudication by a third reviewer (Zhijia Sun). Reasons for exclusion were recorded at the full-text stage.

Data were extracted into a structured form covering study design, participants, exercise or activity exposure, comparator, vaccine, humoral outcome, assessment time, and numerical results. Numerical inputs were checked against source tables, text, and printed figure labels. Printed values were distinguished from statistical reconstructions and readings of plotted coordinates. Exploratory digitization of Hallam et al.’s ELISA fold-change figures was not retained in any revised quantitative analysis because overlapping markers and repeated measurements prevented reliable reconstruction of an independent arm-level estimate. Studies without a compatible numerical estimate were summarized narratively; numerical provenance and exclusions are detailed in **Supplementary Table S7**.

Original investigators were not contacted for missing numerical data. Given the age of several foundational trials and uncertainty about the availability of archived datasets, retrieval of additional numerical information was limited to published reports and their **Supplementary Materials**. No unpublished investigator-supplied values were used. Unresolved numerical ambiguities were excluded from primary analyses or examined only as explicitly labeled sensitivity scenarios; the absence of author contact may have limited the completeness of the quantitative evidence.

### Outcome constructs and harmonization

Humoral outcomes were distinguished by assay and response construct. HAI titers, HAI fold rise, binary thresholds, and ELISA optical-density measures were not treated as interchangeable. H1N1, H3N2, and influenza B were analyzed separately. For the generic influenza B comparisons, a report had to provide one unambiguous B-antigen result; the two B antigens of quadrivalent-vaccine studies were instead reported separately, without selecting one into the generic B synthesis or counting the same participants twice. Peak responses at approximately 3–6 weeks were considered separately from later follow-up.

Postvaccination HAI titers were expressed on a log_2_ scale, so an MD of 1 represents one doubling and 2^MD^ is the corresponding GMT ratio. Kohut (2004) and Woods (2009) provided log_2_ means and standard errors directly. For Lee (2021), log_2_(GMT) was used as the arm mean, and the standard error was approximated from the width of the log_2_-transformed 95% confidence limits divided by 2 × 1.96; the arm standard deviation was obtained by multiplying by the square root of the sample size. The same reconstruction was used for the separately reported nonrandomized Edwards (2010) estimates. The logarithm base in Bohn-Goldbaum (2020) was unstated, so its continuous values were excluded from the primary log_2_ analysis. H3N2 and influenza B were considered only in assumed-log10 sensitivities. H1N1 was excluded from all continuous analyses because the printed control SD of 0.0653 also raised an unresolved decimal-place ambiguity; no replacement SD was assumed. Binary denominators inferred from rounded percentages were identified as reconstructions.

For Lee (2021) fold rise, the published combined-exercise arithmetic mean was compared with control, with standard errors approximated from the printed 95% confidence-limit widths divided by 2 × 1.96. The two B antigens were retained separately. Because its Results text reported B/Brisbane means and SDs that were inconsistent with its fold-rise table, a separate sensitivity used the text-reported values. Rounded or asymmetric confidence limits and discordant dispersion measures limit the precision of these reconstructed single-trial estimates.

Where a trial contributed multiple eligible exercise arms against a shared control, exercise arms were combined before estimation. Means and standard deviations were combined using the standard formula including between-arm mean differences; binary event counts and denominators were summed (41). The shared control was counted once. This applied to the two exercise arms in Elzayat (2021) and Lee (2021), and to the three exercise-intensity arms in the separately reported Edwards (2010) comparison.

### Risk-of-bias assessment

Risk of bias was assessed for humoral results using RoB 2 (2019) for randomized trials, ROBINS-I (2016) for nonrandomized exercise interventions, and ROBINS-E (2024) for observed pre-existing activity or fitness exposures followed by vaccination-response assessment (42–44). Campbell (2010) and Edwards (2010) were described as pseudo-randomized without a demonstrably random sequence and were therefore treated conservatively as nonrandomized; Yang (2007) included additional nonrandomly recruited controls. Kohut (2004) explicitly reported random assignment and was retained as randomized. In Monteiro (2020), supplement randomization did not randomize exercise status; the eligible placebo-arm exercise comparison was treated as observational. Domain meanings and judgment categories were retained separately for each tool. **Supplementary Table S3** reports the least favorable humoral-result judgment for each report and explains the relevant allocation, confounding, missing-data, measurement, and reporting concerns. Small sample size, an active comparator, or figure-only reporting was not itself treated as a risk-of-bias domain.

### Effect measures and synthesis

Binary outcomes were expressed as RRs and continuous HAI titers as MDs on the log_2_ scale. Raw HAI fold-rise MDs were reported only for compatible, unambiguous numerical results; they were not combined with log-fold measures or ELISA optical-density fold change. Positive MDs or RRs above 1 indicate a stronger humoral response in the exercise condition, not necessarily better clinical protection.

Primary syntheses were restricted to randomized exercise comparisons and were conducted separately for acute exercise and chronic training. Pre-existing activity or fitness and nonrandomized interventions were not added to these pools; a single eligible trial was reported as a single-trial estimate. Binary random-effects syntheses used DerSimonian–Laird variance estimation and normal confidence intervals (45, 46). A 0.5 correction was added to all four cells only if a study-level 2 × 2 table had a zero cell after exercise-arm combination; double-zero event comparisons were not assigned an RR. No primary binary comparison required this correction. It was needed for the separately reported Elzayat B/Colorado seroconversion contrast (2/16 versus 0/10) and for the all-event exercise arm in one explicitly hypothetical Hayney H3N2 interpretation. Continuous random-effects syntheses used restricted maximum likelihood; normal intervals were used for two-study pools, with bounded modified Knapp–Hartung intervals as small-k sensitivity analyses. The residual scale was bounded below by 1, and the t distribution used k − 1 degrees of freedom (47).

Heterogeneity was described using I² and τ². A 95% prediction interval was calculated only when at least three studies contributed, using a t distribution with k − 2 degrees of freedom and the sum of between-study variance and pooled-estimate variance (48, 49). With only two or three studies, both heterogeneity and prediction intervals are unstable and cannot establish consistency or predict a precise effect in a new setting. Funnel plots and asymmetry tests were not used because every synthesis contained fewer than ten studies (50).

### Exposure gradients, subgroup and sensitivity analyses

A pooled dose–response analysis requires comparable quantitative exposure levels and their sampling relationships across studies. Those requirements were not met: years or intensity of a training lifestyle and ordinal habitual-activity categories could not be placed on a common dose scale. Consequently, no pooled dose–response curve was fitted; the available ordered exposure contrasts are presented within study only (51, 52).

After separating exposure types and study designs, there were too few comparable trials within age strata for a reliable age interaction or meta-regression. Age-related findings were therefore described without pooling acute exercise in young adults with chronic training in older adults merely to increase subgroup size. Studies enrolling adults aged 50 years or older were not assumed to represent a strictly ≥65-year population. The absence of an interaction analysis does not establish similar effects across ages.

Revision-stage robustness analyses examined leave-one-out estimates within eligible pools, the small-k interval method, nominal versus reconstructed Bohn-Goldbaum binary denominators, the log10 assumption for its H3N2 and influenza B titers, and the addition of Edwards (2010) to an acute mixed-design sensitivity only. The discrepancy between Lee’s table-based and text-based B/Brisbane fold-rise dispersion was also examined. Alternative interpretations of the internally inconsistent Hayney H3N2 seroprotection cells were shown as hypothetical single-trial sensitivities, not adopted as corrections. Woods (2009) used an active flexibility/balance comparator, and omission of that study leaves Kohut (2004) as a single chronic-training trial. Ambiguous Hayney H1N1 fold-rise values and the Hallam digitized readings were excluded rather than assigned a preferred numerical interpretation.

### Certainty of the evidence

Certainty was assessed separately by outcome, antigen, exposure, and design using GRADE. Randomized evidence started at high certainty; nonrandomized evidence started at low certainty, with no upgrading applied (53–56). HAI is direct for the review question of humoral immunogenicity and was not downgraded merely because it does not measure clinical influenza. Risk of bias, inconsistency, imprecision, indirectness, and publication bias were considered separately. Imprecision judgments considered the size of the evidence base, event counts, the range of effects compatible with the interval, and the instability of small-k inference, rather than statistical nonsignificance alone. No validated patient-important threshold for an HAI MD was assumed. Inconsistency was not estimable for a single study, and publication bias was not assumed absent because formal tests were inappropriate. Domain-level downgrades and explanations are given in **Supplementary Table S6**.

### Software

Analyses and figures were generated in Python using NumPy, SciPy, and Matplotlib. The source inputs, transformations, and numerical exclusions are documented in **Supplementary Tables S2** and **S7**. Machine-readable extraction records and analysis code are available from the corresponding authors on reasonable request.

## Results

### Study selection

The recorded selection flow is shown in **Figure 1**. The review dataset contained 22 included reports (57–78). Seven randomized reports supplied at least one eligible primary numerical estimate: Bohn-Goldbaum (2020), Woods (2009), Kohut (2004), Hayney (2014), Elzayat (2021), Ranadive (2014), and Lee (2021) (60, 62, 72–74, 76, 77). Five of these contributed to a synthesis of at least two trials, whereas the remaining primary estimates were single-trial results. Edwards (2010) supplied separate nonrandomized estimates (69). Other findings were retained narratively because of their design, assay, numerical availability, or incompatibility with a specific synthesis; these reasons were not conflated with lack of a biological effect.

**Figure 1:**
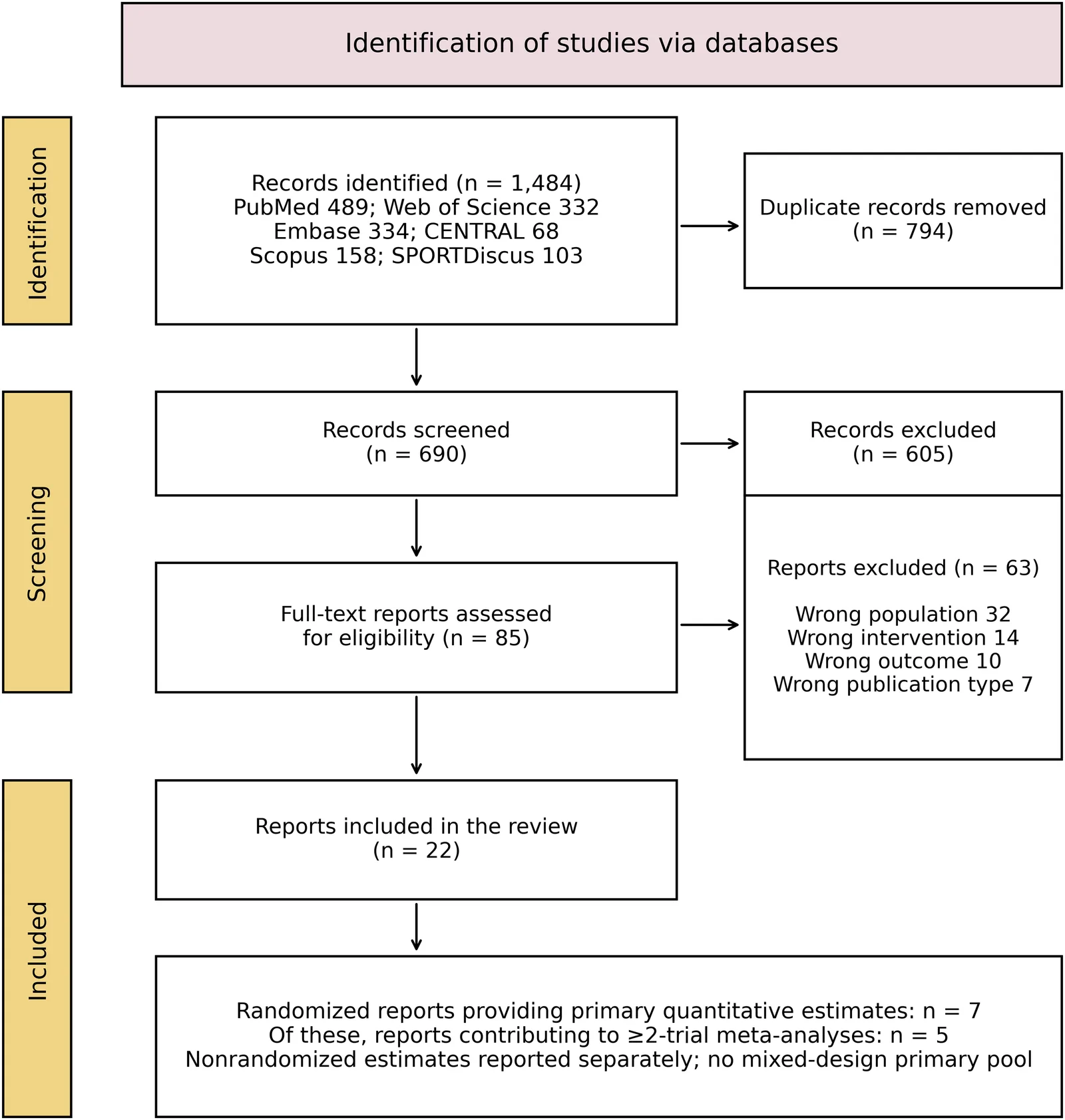
PRISMA 2020 flow diagram of study identification, screening, and inclusion. The recorded database and screening counts are retained through the 22 included reports. The quantitative stage distinguishes seven randomized reports with primary numerical estimates from the five that enter a synthesis of at least two trials. Single-trial estimates are reported separately and nonrandomized comparisons do not enter the primary randomized pools. Reports are not necessarily independent cohorts.

### Characteristics of the included studies

The 22 reports are described in **Tables 1**, **2**. Eleven reports described randomized exercise studies, three described nonrandomized or pseudo-randomized interventions, and eight described observed pre-existing activity or fitness with vaccination-response assessment. They came from the United States (nine), the United Kingdom (six), Brazil (four), Australia (two), and Singapore (one). Monteiro (2020) and Felismino (2021) reported overlapping Brazilian participants, while Hallam (2022) reported more than one influenza experiment. The original sum across reports therefore does not establish the number of unique participants. Outcome-specific sample sizes are reported for each synthesis, and uncertainty in the Bachi (2013) denominator is identified in **Supplementary Table S7**.

**Table 1:** Characteristics of the included reports.

Study	Design	Country	Reported sample/eligible analysis	Population and age	Exercise/physical-activity exposure	Comparator	Reported outcomes	Overall RoB
de Araujo et al. (2015)	Observed exposure with follow-up	Brazil	61 (16/23/22)	Older men 65–85 y	Moderate or intense training lifestyle (17–26 y)	Never trained	HAI titer/seroprotection	High risk (ROBINS-E)
Bachi et al. (2013) (58)	Observed exposure with follow-up	Brazil	110 stated; sex counts imply 90	Older adults 60–85 y	Long-term aerobic + resistance training	Sedentary	Influenza-specific IgG/IgM (ELISA)	High risk (ROBINS-E)
Felismino et al. (2021) (59)	Observed exposure with follow-up	Brazil	80 (31/49)	Older adults	Long-term combined exercise training	Non-practicing	Influenza-specific Ig (ELISA)	High risk (ROBINS-E)
Bohn-Goldbaum et al. (2020) (60)	Randomized trial	Australia	46 (23/23)	Healthy older adults (mean 73)	Acute resistance exercise before vaccination	Seated rest	HAI titer/seroprotection/seroconversion; reactions	Some concerns (RoB 2)
Monteiro et al. (2020) (61)	Observed exercise; supplement randomized	Brazil	84 total; eligible placebo contrast 44 (27/17)	Older adults	Combined exercise training (+ placebo)	Non-practicing (+ placebo)	HAI response; influenza-specific Ig	High risk (ROBINS-E)
Woods et al. (2009) (62)	Randomized trial	USA	144 (74/70)	Sedentary older adults	10-month cardiovascular training	Flexibility/balance (active)	HAI titer/seroprotection	High risk (RoB 2)
Long et al. (2012) (63)	Randomized trial	UK	120	Young and older adults	45-min brisk walk before vaccination	Seated rest	HAI titer; responder status	Some concerns (RoB 2)
Hallam et al. (2022) (64)	Randomized trial	USA	16 + 26 (influenza)	Young and older adults	45- or 90-min aerobic exercise after vaccination	No exercise	Influenza-specific IgG OD fold change	High risk (RoB 2)
Campbell et al. (2010) (65)	Pseudo-randomized intervention	UK	155	Healthy young adults	Eccentric exercise at 0/6/48 h before vaccination	Resting control	HAI titer; IFN-γ	Serious risk (ROBINS-I)
Keylock et al. (2007) (66)	Observed exposure with follow-up	USA	26 (13/13)	Older adults 60–76 y	High cardiorespiratory fitness	Low fitness	HAI titer/fold/seroconversion	High risk (ROBINS-E)
Edwards et al. (2006) (67)	Randomized trial	UK	60 total; 40 exercise/rest	Healthy young adults (mean 22)	Acute dynamic exercise before vaccination	Resting control	HAI titer	High risk (RoB 2)
Edwards et al. (2007) (68)	Randomized trial	UK	60 (40/20)	Healthy young adults	Eccentric exercise 6 h before vaccination	Resting control	HAI titer; IFN-γ	Some concerns (RoB 2)
Edwards et al. (2010) (69)	Pseudo-randomized intervention	UK	158	Healthy young adults	Eccentric exercise 60/85/110% 1RM before vaccination	Resting control	HAI titer/GMT/fold change	Serious risk (ROBINS-I)
Ayling et al. (2018) (70)	Observed exposure with follow-up	UK	138 (122 at 16 wk)	Community older adults 65–85 y (mean 73)	Habitual physical activity (daily steps)	Lower activity (continuous)	HAI seroprotection; strain-specific IgG	High risk (ROBINS-E)
Kohut et al. (2002) (71)	Observed exposure with follow-up	USA	56 (15/25/16)	Older adults ≥62 y	Active, moderately active, and sedentary habitual activity	Sedentary	Influenza-specific IgG/IgM; influenza-like illness	High risk (ROBINS-E)
Kohut et al. (2004) (72)	Randomized trial	USA	27 (14/13)	Older adults ≥64 y	10-month aerobic exercise	Non-training control	HAI titer/fold change	Some concerns (RoB 2)
Hayney et al. (2014) (73)	Randomized trial	USA	98 (47 ex/51 waitlist)	Adults ≥50 y	8-week exercise program	Waitlist	HAI titer/fold/seroprotection/seroconversion; nasal IgA	Some concerns (RoB 2)
Elzayat et al. (2021) (74)	Randomized trial	USA	29 randomized; 26 at 6 weeks (16/10)	Older adults ≥65 y	Eccentric exercise (same/opposite arm) before vaccination	No exercise	HAI titer/seroprotection/seroconversion; IFN-γ	Some concerns (RoB 2)
Sadarangani et al. (2022) (75)	Observed exposure with follow-up	Singapore	220	Older adults ≥65 y	Graded habitual PA (RAPA categories)	Rare activity	HAI fold rise/seroconversion	High risk (ROBINS-E)
Ranadive et al. (2014) (76)	Randomized trial	USA	55 (27/28)	Older adults 55–75 y	Acute aerobic exercise before vaccination	No exercise	HAI titer/seroprotection	Some concerns (RoB 2)
Lee et al. (2021) (77)	Randomized trial	Australia	57 (19/19/19)	Healthy young adults	Arm or leg exercise before vaccination	No exercise	HAI titer/GMT/fold change	Some concerns (RoB 2)
Yang et al. (2007) (78)	Nonrandomized/mixed-control intervention	USA	50 (27/23)	Older adults	5-month Taiji/Qigong	Waitlist	HAI response/seroprotection	Serious risk (ROBINS-I)

RoB, risk of bias; HAI, hemagglutination inhibition; Ig, immunoglobulin; IFN-γ, interferon gamma; PA, physical activity; RAPA, Rapid Assessment of Physical Activity; RCT, randomized controlled trial; 1RM, one-repetition maximum. Sample sizes are report- or contrast-specific and must not be summed as unique participants. Bachi reports inconsistent denominators; Monteiro and Felismino contain overlapping participants; Hallam contains separate influenza experiments. The overall rating is the least favorable humoral-result judgment, not a composite of humoral, cell-mediated, and clinical outcomes. Native tool categories are retained. Reported outcomes may include nonhumoral endpoints, which were not substituted for eligible humoral outcomes.

**Table 2:** Detailed exercise/exposure characteristics of the included studies.

Study	Exposure type	Mode	Dose/intensity	Duration	Frequency	Timing relative to vaccination	Influenza vaccine
de Araujo et al. (2015)	Observed training lifestyle	Mixed training lifestyle	Moderate/intense	17–26 years	Habitual	Habitual	Seasonal trivalent
Bachi et al. (2013) (58)	Observed training lifestyle	Aerobic + resistance	1 h/session	≥12 months	4/week	Habitual	Seasonal trivalent
Felismino et al. (2021) (59)	Observed training lifestyle	Combined exercise	60–75 min	≥12 months	3/week	Habitual	Seasonal trivalent
Bohn-Goldbaum et al. (2020) (60)	Acute exercise	Resistance	Moderate, 45 min	Single session	Once	Immediately before	Seasonal
Monteiro et al. (2020) (61)	Observed training lifestyle	Combined exercise	60–75 min	≥12 months	3/week	Habitual	Seasonal trivalent
Woods et al. (2009) (62)	Long-term training	Cardiovascular	60-70% VO_2_max, 45–60 min	10 months	3/week	Chronic before	Seasonal trivalent
Long et al. (2012) (63)	Acute exercise	Brisk walk	>55% HRmax, 45 min	Single session	Once	Immediately before	Half-dose seasonal
Hallam et al. (2022) (64)	Acute exercise	Aerobic (cycle/walk)	45 or 90 min; ~65–70% HRmax	Single session	Once	After vaccination	Monovalent H1N1/seasonal
Campbell et al. (2010) (65)	Acute exercise	Eccentric (biceps/deltoid)	85% 1RM	Single session	Once	0, 6 or 48 h before	Seasonal trivalent
Keylock et al. (2007) (66)	Fitness (VO_2_max)	Cardiorespiratory fitness	46.8 vs 21.1 mL/kg/min	Chronic	Habitual	Habitual	Seasonal trivalent
Edwards et al. (2006) (67)	Acute exercise	Dynamic aerobic	Moderate, 45 min	Single session	Once	Immediately before	Seasonal trivalent
Edwards et al. (2007) (68)	Acute exercise	Eccentric (arm)	Vigorous	Single session	Once	6 h before	Seasonal trivalent
Edwards et al. (2010) (69)	Acute exercise	Eccentric (arm)	60/85/110% 1RM	Single session	Once	Immediately before	Half-dose seasonal
Ayling et al. (2018) (70)	Habitual PA	Pedometer steps/day	Continuous (observed)	Diary period	Daily living	Habitual	Seasonal trivalent
Kohut et al. (2002) (71)	Habitual PA	Habitual activity level	Active/moderate/sedentary	Prior year	Habitual	Habitual	Seasonal trivalent
Kohut et al. (2004) (72)	Long-term training	Aerobic	65-75% HRR, 25–30 min	10 months	3/week	Chronic before	Seasonal trivalent
Hayney et al. (2014) (73)	Exercise training	Aerobic (MEPARI)	Borg RPE 12–16; 45 min/day home practice	8 weeks	Weekly 2.5-h group session; daily home practice	Vaccinated during week 6	Seasonal trivalent
Elzayat et al. (2021) (74)	Acute exercise	Eccentric (biceps)	Vigorous	Single session	Once	Immediately before	Seasonal quadrivalent
Sadarangani et al. (2022) (75)	Habitual PA	RAPA activity categories	Rare→vigorous	Habitual	Habitual	Habitual	Seasonal
Ranadive et al. (2014) (76)	Acute exercise	Aerobic	55–65% HRmax, 45 min	Single session	Once	Immediately before	Seasonal trivalent
Lee et al. (2021) (77)	Acute exercise	Elastic-band arm/body-weight leg	Moderate, 15 min	Single session	Once	Immediately before	Quadrivalent; 0.25 mL (half-dose)
Yang et al. (2007) (78)	Mind-body exercise	Taiji/Qigong	60 min	5 months	3/week	Started before	Seasonal trivalent

HRmax, maximum heart rate; HRR, heart-rate reserve; NR, not reported; RPE, rating of perceived exertion; RAPA, Rapid Assessment of Physical Activity; VO_2_max, maximal oxygen uptake; 1RM, one-repetition maximum. “Habitual” denotes an observed pre-existing exposure rather than an assigned intervention. Hallam’s 45 and 90 minutes denote exercise duration after vaccination. Reduced-dose vaccination in Long, Edwards (2010), and Lee is distinguished from standard-dose vaccination.

Ten reports examined acute exercise near vaccination; three randomized reports examined structured chronic training; four observational reports compared pre-existing training lifestyles; three examined habitual activity; one examined fitness; and one nonrandomized study examined Taiji/Qigong. Age definitions varied, and several studies included middle-aged rather than exclusively older adults. Hallam (2022) evaluated exercise lasting 45 or 90 minutes after vaccination, rather than vaccination-to-exercise delays of 45 or 90 minutes. Woods (2009) used flexibility/balance training as an active comparator. Reduced vaccine doses were used in Long (2012), Edwards (2010), and Lee (2021), limiting extrapolation of those results to routine-dose vaccination.

Primary randomized estimates, separated into acute and chronic exercise and explicitly distinguishing pooled from single-trial results, are summarized in **Table 3**.

**Table 3:** Primary randomized estimates, separated by acute exercise and chronic training.

Outcome/exposure	k (participants)	Effect (95% CI)	I²	p	95% PI	GRADE
Seroprotection – H1N1 Acute exercise	3 (125)	RR 0.88 (0.65 to 1.21)	0%	0.434	0.12 to 6.72	Low
Seroprotection – H1N1 Chronic exercise	1 (98)	RR 0.96 (0.80 to 1.15)	—	0.653	—	Low
Seroprotection – H3N2 Acute exercise	3 (126)	RR 1.04 (0.91 to 1.20)	0%	0.567	0.42 to 2.59	Low
Seroprotection – Influenza B Acute exercise	1 (55)	RR 0.35 (0.04 to 3.12)	—	0.344	—	Very low
Seroconversion – H1N1 Acute exercise	2 (70)	RR 1.30 (0.58 to 2.92)	0%	0.530	—	Very low
Seroconversion – H1N1 Chronic exercise	1 (98)	RR 1.04 (0.66 to 1.62)	—	0.878	—	Low
Seroconversion – H3N2 Acute exercise	2 (71)	RR 0.84 (0.50 to 1.40)	0%	0.496	—	Very low
Seroconversion – H3N2 Chronic exercise	1 (98)	RR 1.09 (0.81 to 1.45)	—	0.579	—	Low
Seroconversion – Influenza B Acute exercise	1 (45)	RR 0.24 (0.03 to 1.98)	—	0.184	—	Very low
Seroconversion – Influenza B Chronic exercise	1 (98)	RR 0.94 (0.60 to 1.48)	—	0.800	—	Low
HAI titer – H1N1 Acute exercise	1 (57)	MD 0.52 (-0.51 to 1.56)	—	0.322	—	Very low
HAI titer – H1N1 Chronic exercise	2 (171)	MD 0.50 (-0.64 to 1.64)	68%	0.392	—	Very low
HAI titer – H3N2 Acute exercise	1 (57)	MD -0.47 (-1.38 to 0.44)	—	0.314	—	Very low
HAI titer – H3N2 Chronic exercise	2 (171)	MD 0.27 (-0.17 to 0.72)	0%	0.233	—	Very low
HAI titer – Influenza B Chronic exercise	2 (171)	MD -0.14 (-0.64 to 0.36)	0%	0.593	—	Very low

k is the number of independent randomized comparisons; k = 1 denotes a single-trial estimate, not a meta-analysis. RRs above 1 and positive MDs indicate a stronger humoral response. Binary pools use DerSimonian–Laird random effects; two-trial continuous pools use REML with normal confidence intervals. Bounded modified Knapp–Hartung sensitivities are reported in **Supplementary Table S4**. A prediction interval is shown only for k ≥3. Distinct B antigens from quadrivalent vaccines, unclear log-scale values, nonrandomized studies, and ambiguous source cells are not entered into these primary pools. CI, confidence interval; HAI, hemagglutination inhibition; MD, mean difference in log_2_ doublings; PI, prediction interval; RR, risk ratio.

### Risk of bias

Design-specific assessments are shown in **Figure 2** and **Table 4**, with supporting judgments in **Supplementary Table S3**. Among 11 reports assessed with RoB 2, eight had some concerns and three were at high risk for the humoral results considered. All three nonrandomized intervention reports were at serious risk with ROBINS-I. All eight observational reports had at least one high-risk humoral result with ROBINS-E. For Keylock (2007), the unadjusted seroconversion comparisons had high risk from confounding, whereas partially adjusted continuous-response analyses retained some concerns. These categories and domain numbers are tool-specific; no cross-tool domain percentages were calculated.

**Figure 2:**
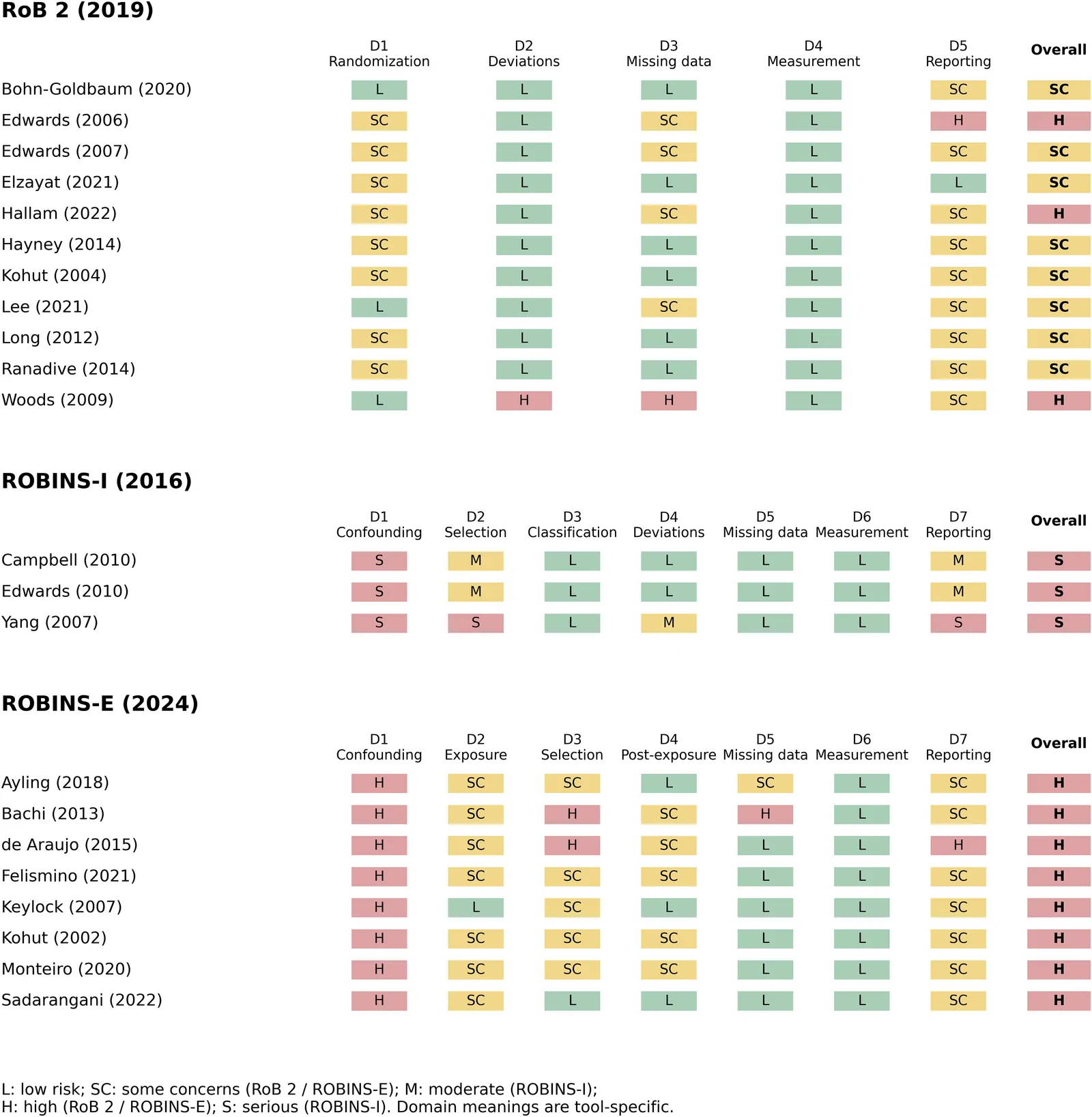
Risk of bias in the included studies. Tool-specific risk-of-bias displays for humoral results: RoB 2 (2019), ROBINS-I (2016), and ROBINS-E (2024). Each tool has its own domain headings and judgment categories; no domain percentages or judgments are aggregated across tools. The final column shows the least favorable humoral-result judgment for the report. L, low risk; SC, some concerns (RoB 2/ROBINS-E); M, moderate (ROBINS-I); H, high (RoB 2/ROBINS-E); S, serious (ROBINS-I).

**Table 4:** Overall risk of bias for humoral results in the included reports.

Report	Design of exercise contrast	Tool	Overall humoral RoB	Principal reason
de Araujo et al. (2015)	Observed pre-existing activity/fitness exposure with postvaccination follow-up	ROBINS-E (2024)	High risk	Training groups were self-selected and recruited from different community and sport settings.
Bachi et al. (2013) (58)	Observed pre-existing activity/fitness exposure with postvaccination follow-up	ROBINS-E (2024)	High risk	Exercise and sedentary groups were non-randomized and recruited from different source populations; baseline BMI differed and no confounding-adjusted effect was reported.
Felismino et al. (2021) (59)	Observed pre-existing activity/fitness exposure with postvaccination follow-up	ROBINS-E (2024)	High risk	The training exposure was established before recruitment and was not allocated by investigators.
Bohn-Goldbaum et al. (2020) (60)	Randomized controlled trial	RoB 2 (2019)	Some concerns	Humoral-result selection concerns; ambiguous numerical cells are handled separately.
Monteiro et al. (2020) (61)	Observed pre-existing activity/fitness exposure with postvaccination follow-up	ROBINS-E (2024)	High risk	Pre-existing exercise exposure was not randomized; only supplementation was randomized.
Woods et al. (2009) (62)	Randomized controlled trial	RoB 2 (2019)	High risk	Postrandomization exclusions and missing outcomes; not the active comparator itself.
Long et al. (2012) (63)	Randomized controlled trial with young and older strata	RoB 2 (2019)	Some concerns	The CONSORT diagram reports 122 randomized participants, but sequence generation and allocation concealment were not described.
Hallam et al. (2022) (64)	Randomized controlled experiments	RoB 2 (2019)	High risk	Unclear allocation details and postrandomization exclusions in the monovalent experiment; multiple result choices.
Campbell et al. (2010) (65)	Nonrandomized or quasi-randomized exercise intervention	ROBINS-I (2016)	Serious risk	Pseudo-random allocation; confounding and allocation selection not resolved.
Keylock et al. (2007) (66)	Observed pre-existing activity/fitness exposure with postvaccination follow-up	ROBINS-E (2024)	High risk	Unadjusted seroconversion ratios in selected fitness groups; separate continuous-model adjustment does not control confounding in these binary estimates.
Edwards et al. (2006) (67)	Three-arm randomized controlled trial	RoB 2 (2019)	High risk	Random allocation was stated, but sequence generation and concealment were not described.
Edwards et al. (2007) (68)	Randomized controlled trial	RoB 2 (2019)	Some concerns	A pre-determined restricted random assignment was used, but allocation concealment was not described.
Edwards et al. (2010) (69)	Nonrandomized or quasi-randomized exercise intervention	ROBINS-I (2016)	Serious risk	Selection of a sex-specific H3N2 result among multiple strain and timepoint analyses, with incompletely reported allocation procedures.
Ayling et al. (2018) (70)	Observed pre-existing activity/fitness exposure with postvaccination follow-up	ROBINS-E (2024)	High risk	The extracted physical-activity associations are unadjusted correlations, leaving age, prior vaccination, baseline immunity, health, and correlated behaviors insufficiently controlled.
Kohut et al. (2002) (71)	Observed pre-existing activity/fitness exposure with postvaccination follow-up	ROBINS-E (2024)	High risk	Pre-existing activity categories; baseline immunity and lifestyle confounding not adequately controlled.
Kohut et al. (2004) (72)	Randomized exercise-training trial (random assignment explicitly stated)	RoB 2 (2019)	Some concerns	Random assignment stated; sequence/concealment and prespecified analysis details incomplete.
Hayney et al. (2014) (73)	Three-arm randomized controlled trial	RoB 2 (2019)	Some concerns	Random assignment to exercise, MBSR, or waitlist was reported, but sequence generation and concealment were not described in this report.
Elzayat et al. (2021) (74)	Three-arm randomized controlled trial	RoB 2 (2019)	Some concerns	The random sequence was generated in Excel, but allocation concealment was not reported.
Sadarangani et al. (2022) (75)	Observed pre-existing activity/fitness exposure with postvaccination follow-up	ROBINS-E (2024)	High risk	The multivariable models adjusted many measured characteristics, including baseline HAI and prior vaccination, but important residual health-behavior and frailty confounding remains.
Ranadive et al. (2014) (76)	Randomized controlled trial	RoB 2 (2019)	Some concerns	Participants were randomized to exercise or no exercise, but sequence generation and concealment were not described.
Lee et al. (2021) (77)	Three-arm randomized controlled trial	RoB 2 (2019)	Some concerns	Unexplained exclusion of one pre-leg participant and incomplete prespecification of pooled versus arm-specific analyses.
Yang et al. (2007) (78)	Nonrandomized or quasi-randomized exercise intervention	ROBINS-I (2016)	Serious risk	Nine additional controls were recruited nonrandomly; age/recruitment/attention imbalance.

RoB 2 applies only to randomized exercise trials; ROBINS-I (2016) to nonrandomized exercise interventions; ROBINS-E (2024) to observed pre-existing activity/fitness exposure comparisons. The most unfavorable humoral-result judgment is shown, with supporting outcome-level records and rationale in **Supplementary Table S3** and the reproducibility data. Domain numbers and categories retain their tool-specific meaning. Small sample size, an active comparator, vaccine dose, or figure-only reporting alone does not determine a bias rating.

Randomized-trial concerns chiefly involved inadequately described allocation, postrandomization exclusions in some trials, and selection among multiple antibody metrics, strains, timepoints, and subgroup analyses. The pseudo-randomized studies did not establish a genuinely random allocation sequence, while Yang (2007) mixed randomized and additional nonrandomized controls. For observational exposures, health, frailty, body composition, prior vaccination, baseline immunity, and correlated lifestyle factors could confound associations. Objective laboratory assays reduced some measurement concerns but did not resolve allocation or confounding problems. Numerical reporting limitations were recorded separately from the bias judgments.

### Seroprotection

For acute exercise, three randomized trials contributed to each influenza A seroprotection synthesis (**Figure 3**; **Table 3**) (60, 74, 76). The H1N1 RR was 0.88 (95% CI 0.65–1.21; 125 participants; I² = 0%), and the H3N2 RR was 1.04 (0.91–1.20; 126 participants; I² = 0%). Neither estimate established benefit or equivalence. Chronic-training H1N1 seroprotection was supported by Hayney (2014) alone (RR 0.96, 0.80–1.15; 98 participants) (73). The H3N2 seroprotection cells in that report were not used in the primary analysis because the printed column labels and denominators were inconsistent.

**Figure 3:**
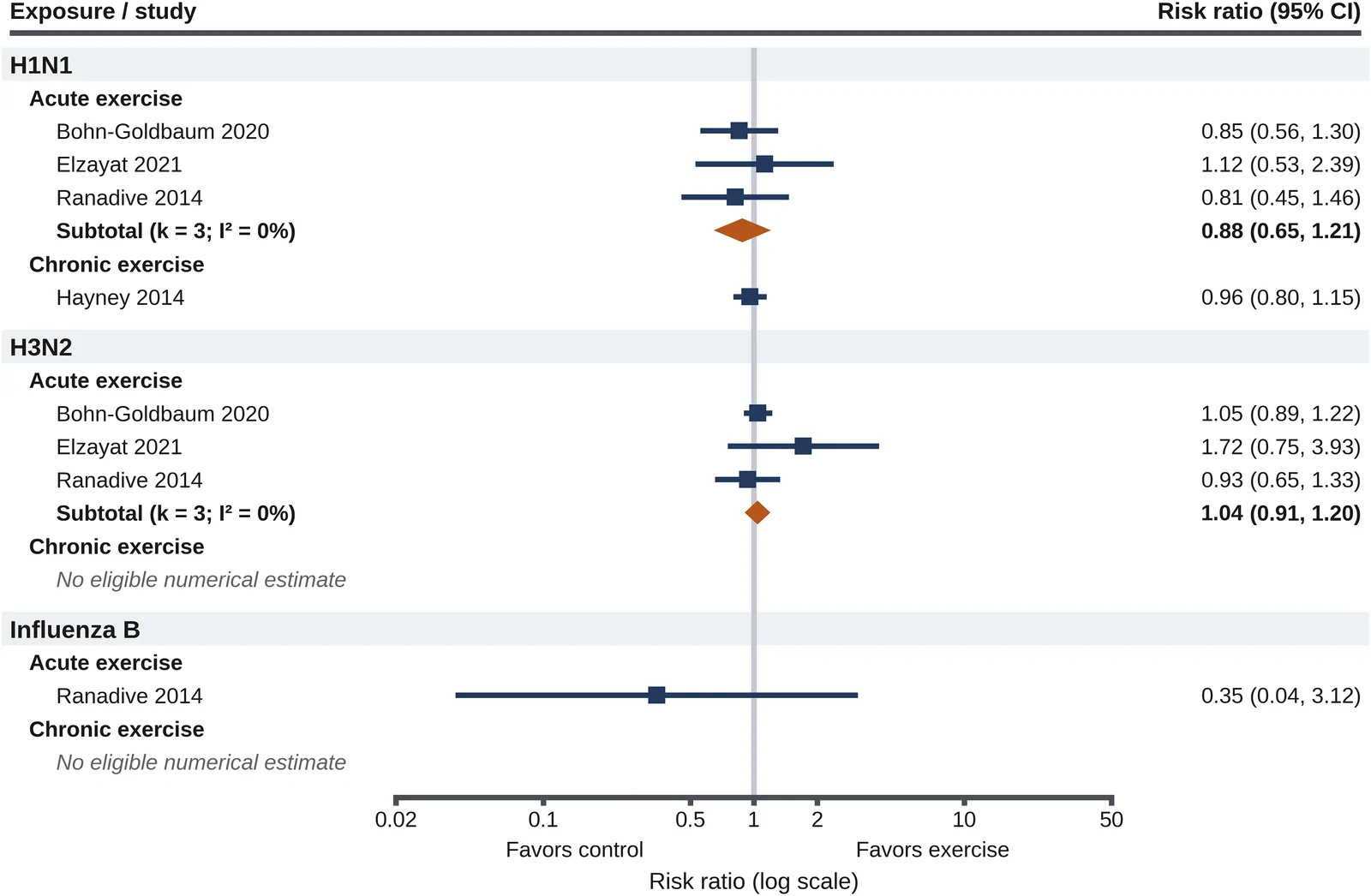
HAI seroprotection, separated by acute exercise and chronic training. Randomized-trial risk ratios for postvaccination HAI titer ≥1:40 at approximately 3–6 weeks. H1N1, H3N2, and the generic influenza B comparison are shown separately, with distinct acute and chronic strata. A diamond denotes a within-stratum random-effects subtotal only when at least two trials contribute; a single square is an individual trial estimate. No overall acute-plus-chronic estimate is calculated. Distinct B-antigen Elzayat results and ambiguous source cells are not selected into the generic B analysis. RR >1 favors a stronger humoral response with exercise. CI, confidence interval; HAI, hemagglutination inhibition; RR, risk ratio.

The generic influenza B acute seroprotection result was a single-trial estimate from Ranadive (2014): 1/27 versus 3/28, RR 0.35 (0.04–3.12) (76). Elzayat (2021) reported two distinct B antigens and was not represented by selecting one of them into that generic result: B/Phuket seroprotection was 2/16 versus 4/10, whereas B/Colorado had no events in either group (0/16 versus 0/10) (74). These antigen-specific results are listed separately in **Supplementary Table S2**. No unambiguous chronic-training generic B seroprotection estimate was available at the peak assessment. Sparse events preclude an inference of harm.

### Seroconversion

Two acute randomized trials provided H1N1 and H3N2 seroconversion data (**Figure 4**; **Table 3**) (60, 74). RRs were 1.30 (95% CI 0.58–2.92; 70 participants) for H1N1 and 0.84 (0.50–1.40; 71 participants) for H3N2; I² was 0% in both small syntheses. The generic B acute estimate came from Bohn-Goldbaum (2020) alone (RR 0.24, 0.03–1.98; 45 participants). Chronic-training estimates came from Hayney (2014) alone: H1N1 RR 1.04 (0.66–1.62), H3N2 1.09 (0.81–1.45), and influenza B 0.94 (0.60–1.48), each with 98 participants (73). The separate Elzayat B-antigen estimates, including the corrected zero-cell B/Colorado contrast, are reported in **Supplementary Table S2** rather than pooled with a different B antigen.

**Figure 4:**
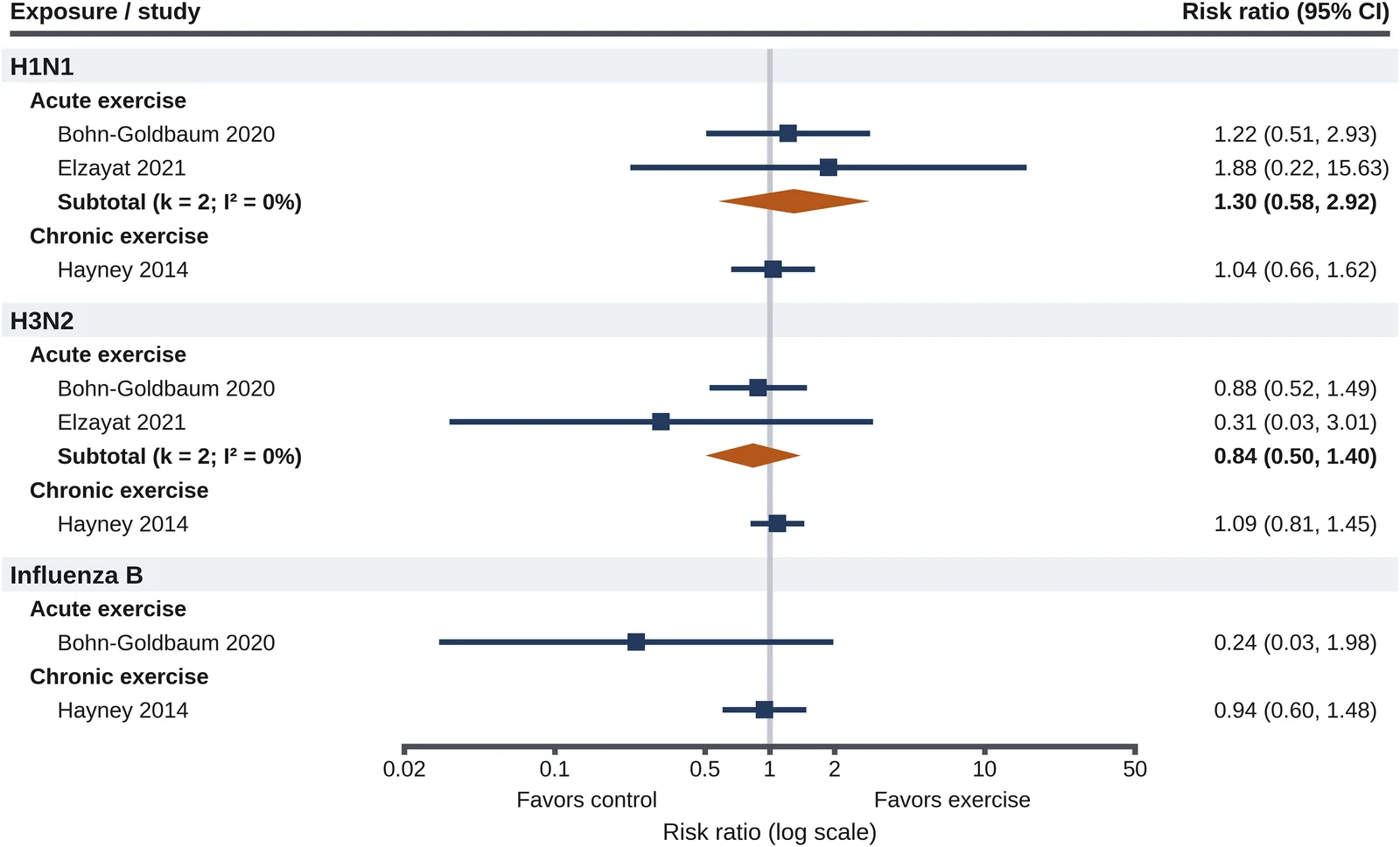
HAI seroconversion, separated by acute exercise and chronic training. Randomized-trial risk ratios for a ≥4-fold HAI titer rise at approximately 3–6 weeks, with separate antigen and exposure strata. Diamonds denote random-effects subtotals for at least two trials; single trials are not portrayed as pooled evidence. Generic influenza B results are not mixed with a selected B antigen from a quadrivalent vaccine. No primary contrast required continuity correction after exercise-arm combination. The separately reported Elzayat B/Colorado contrast requiring correction is listed in **Supplementary Table S2**. CI, confidence interval; HAI, hemagglutination inhibition; RR, risk ratio.

### Post-vaccination HAI antibody titer

Primary log_2_ titer analyses are shown in **Figure 5** and **Table 3**. Chronic training was represented by Kohut (2004) and Woods (2009), totaling 171 participants: H1N1 MD 0.50 log_2_ units (95% CI −0.64 to 1.64; I² = 68%), H3N2 0.27 (−0.17 to 0.72; I² = 0%), and influenza B −0.14 (−0.64 to 0.36; I² = 0%) (62, 72). These estimates correspond to GMT ratios of approximately 1.41, 1.21, and 0.91, respectively, but their intervals do not establish a training effect. Acute primary titer estimates came from Lee (2021) alone (57 participants): H1N1 MD 0.52 (−0.51 to 1.56) and H3N2 −0.47 (−1.38 to 0.44) (77). Its two B lineages are reported separately. Bohn-Goldbaum’s unresolved H1N1 continuous result, its assumed-log10 H3N2/B estimates, and nonrandomized Edwards estimates do not enter these primary randomized results.

**Figure 5:**
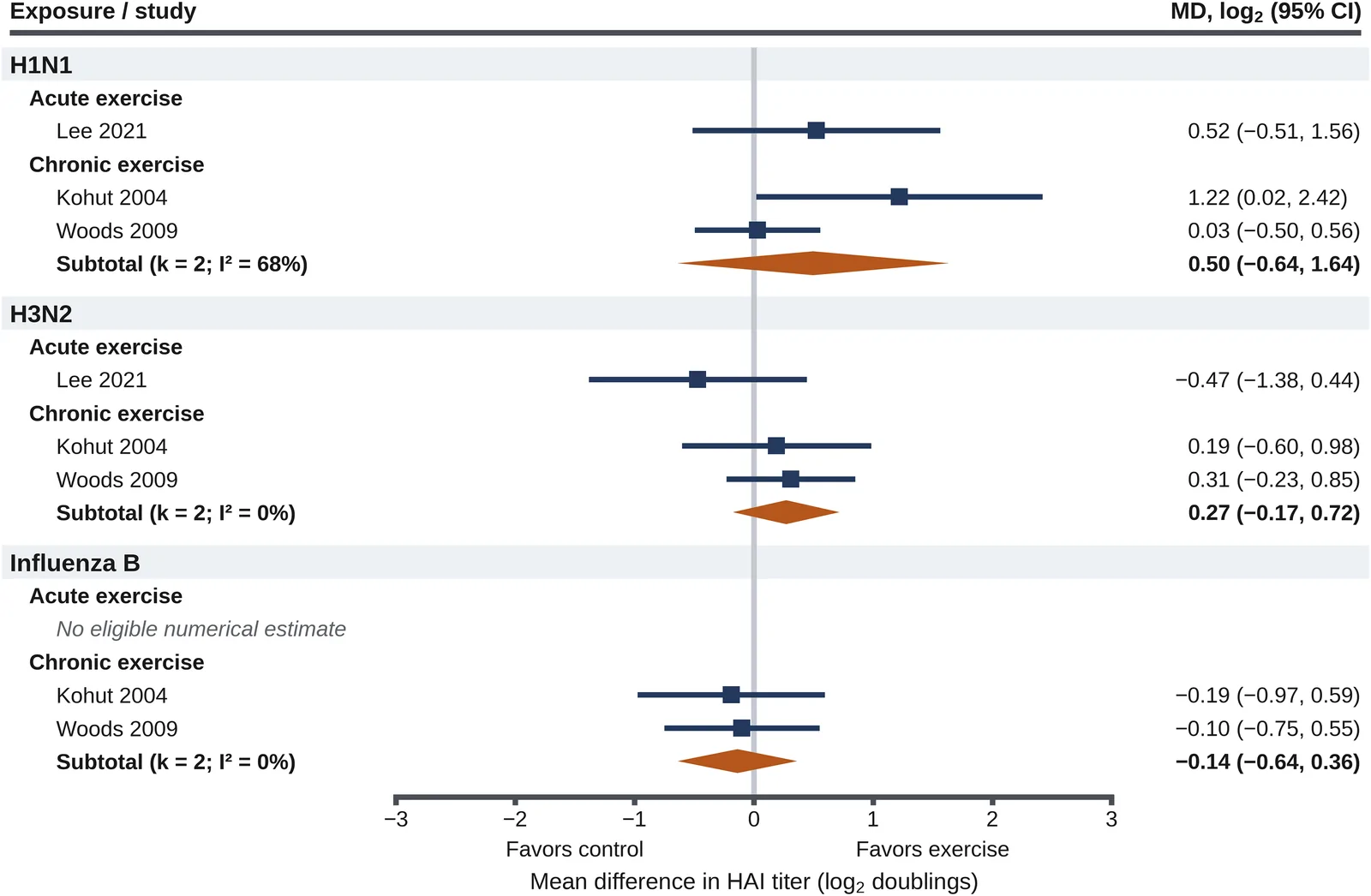
Postvaccination HAI titers on a log_2_ scale, separated by acute exercise and chronic training. Randomized-trial MDs in log_2_ HAI titers at approximately 3–4 weeks. An MD of 1 represents one doubling. Chronic-training subtotals combine Kohut (2004) and Woods (2009) with REML and normal confidence intervals; bounded modified Knapp–Hartung sensitivity intervals are reported in **Supplementary Table S4**. Acute primary estimates come from Lee (2021) alone. Woods used an active flexibility/balance comparator. Bohn-Goldbaum’s H1N1 continuous result is excluded because both scale and dispersion remain unresolved; its H3N2/B assumed-log10 values and the nonrandomized Edwards estimates appear only in separate sensitivity or single-study analyses. No primary titer stratum contains three trials, so no prediction interval is shown. CI, confidence interval; MD, mean difference; REML, restricted maximum likelihood.

The H1N1 contrast in Kohut (2004) was larger than that in Woods (2009), but this is a comparison of two small and clinically different trials, not a demonstrated moderator effect (62, 72). Woods used an active comparator, whereas Kohut used a nontraining comparator. The chronic-training intervals became substantially wider under bounded modified Knapp–Hartung inference (**Supplementary Table S4**), illustrating the limited information available with only two trials.

### HAI antibody fold rise

Raw HAI fold-rise estimates are presented individually in **Supplementary Figure S1** and **Supplementary Table S2**, without pooling acute and chronic exposures (73, 77). In Lee (2021), the acute-exercise MD was −0.60 (95% CI −17.95 to 16.75) for H1N1 and 0.00 (−11.67 to 11.67) for H3N2. Its separately reported B-antigen estimates were 5.00 (−2.76 to 12.76) for B/Phuket/3073/2013 and 5.50 (0.92 to 10.08) for B/Brisbane/60/2008, using the means and confidence limits in **Table 3**. The B/Brisbane interval included the null when the discordant means and SDs in the article’s Results text were used instead (MD 5.60, −0.86 to 12.06; **Supplementary Table S4**). Thus, the apparent benefit depended on the source dispersion representation. Hayney (2014) supplied chronic-training H3N2 and influenza B estimates, but its H1N1 values were excluded: the printed means of 0.65 and 1.43 are incompatible with raw fold ratios when 21/47 and 22/51 participants, respectively, achieved a ≥4-fold rise. No alternative scale or corrected values were assumed.

### Nonpooled findings and observational exposure gradients

Randomized results not suitable for pooling, nonrandomized interventions, and observed exposures are summarized separately in **Supplementary Table S5**; within-study exposure gradients are displayed in **Supplementary Figure S2**. Hallam (2022) reported greater ELISA antibody responses after 90-minute postvaccination exercise, but those optical-density outcomes were not combined with HAI titers or HAI fold rise (64). The Taiji/Qigong comparison was nonrandomized and did not establish a strain-specific randomized effect (78). Observational reports described selected antibody associations, but not a uniform improvement across antibody classes or strains (57–59, 61, 66, 70, 71, 75).

In de Araujo (2015), unadjusted six-week seroprotection estimates favored the moderately and intensely trained groups for H3N2 (RR 1.38, 95% CI 0.87–2.17; and 1.52, 0.98–2.34) and influenza B (1.83, 0.83–4.01; and 2.05, 0.95–4.42), but not clearly for H1N1; all intervals included 1 (57). In Sadarangani (2022), adjusted H3N2 fold-rise ratios relative to rare activity were 2.04, 2.52, 2.78, and 2.53 across increasing RAPA categories (reported linear-trend p = 0.015), without a corresponding gradient for H1N1 or influenza B (75, 79). These associations remain susceptible to healthy-user bias: more active individuals may also have better sleep and diet, fewer comorbidities, and less frailty. They do not identify the causal effect of prescribing exercise.

In the small Keylock (2007) fitness comparison, unadjusted seroconversion RRs were 2.67 (95% CI 0.90–7.86; 8/13 versus 3/13) for H1N1 and 3.50 (0.89–13.78; 7/13 versus 2/13) for influenza B (66). These unadjusted ratios were judged at high risk of confounding; covariate adjustment of separate continuous models did not adjust the binary comparisons. In Kohut (2002), the influenza-like-illness contrast of 3/25 versus 1/15 (RR 1.80, 0.21–15.78) compared moderately active with sedentary participants, not the vigorous-activity group; it was a clinical observation rather than a humoral outcome and was not pooled (71). Observational certainty was very low after considering residual confounding, selection, and imprecision; no exposure category was interpreted as a randomized exercise prescription.

### Subgroup analysis

An age interaction could not be examined reliably after separating intervention duration, design, and numerical eligibility. Age coincided with differences in exposure and study design, and most strata contained only one or two comparable reports. **Supplementary Table S5** retains the age context and explains why no between-age effect was estimated.

### Sensitivity analyses

Sensitivity results are presented in **Supplementary Table S4**. Leave-one-out analyses of the acute influenza A seroprotection pools and the acute seroconversion pools did not establish a benefit. For chronic H1N1 titer, however, omission of Woods (2009) leaves the positive single-trial Kohut contrast; omission of Kohut leaves the near-null Woods contrast. Thus, robustness cannot be summarized as independence from any single study. Bounded modified Knapp–Hartung intervals were much wider than normal intervals in the two-trial syntheses and are important to the interpretation of uncertainty.

Using nominal 23/23 Bohn-Goldbaum denominators rather than those reconstructed from the printed percentages did not resolve the overall uncertainty. Assuming base-10 logarithms allowed additional H3N2 and influenza B titer sensitivities but did not confirm that scale; the unresolved H1N1 continuous result was excluded. The Lee B/Brisbane text-SD sensitivity illustrates additional uncertainty from inconsistent source dispersion. The Edwards (2010) comparison and alternative interpretations of the Hayney H3N2 table cells were also treated as sensitivities, not incorporated into primary randomized evidence. Woods (2009) reported seroprotection at 24 weeks in printed figure labels; those later responses were not mixed with the peak 3–6-week window (62).

### Reporting bias

All pooled analyses contained fewer than ten trials, so funnel plots and asymmetry tests were not appropriate (50). Reporting bias could neither be established nor excluded statistically. Selective result reporting was considered within the applicable risk-of-bias tool, while figure-only results, unclear statistical labels, inconsistent table cells, and the lack of investigator contact for additional data limited quantitative availability. Lack of extractable numbers was not interpreted as a null result.

### Certainty of the evidence

Certainty in the primary randomized comparisons was low or very low (**Table 5**; **Supplementary Table S6**). The principal limitations were trial-level bias and imprecision, with an additional inconsistency downgrade for chronic-training H1N1 titers. No estimate was downgraded solely because HAI is an immunogenicity rather than a clinical outcome. Nonrandomized estimates started at low certainty and were considered separately; they did not inherit the high starting level of randomized evidence. Sparse evidence and the inability to assess publication bias remained limitations, not evidence that bias was absent.

**Table 5:** Summary of findings (GRADE) for the primary randomized comparisons.

Outcome/exposure	k (participants)	Effect (95% CI)	GRADE certainty	Interpretation/illustrative absolute risk
Seroprotection – H1N1 Acute	3 (125)	RR 0.88 (0.65 to 1.21)	Low	Control: 576/1,000; exercise: 509/1,000 (372 to 696).
Seroprotection – H1N1 Chronic	1 (98)	RR 0.96 (0.80 to 1.15)	Low	Control: 843/1,000; exercise: 809/1,000 (674 to 971).
Seroprotection – H3N2 Acute	3 (126)	RR 1.04 (0.91 to 1.20)	Low	Control: 738/1,000; exercise: 769/1,000 (668 to 884).
Seroprotection – Influenza B Acute	1 (55)	RR 0.35 (0.04 to 3.12)	Very low	Control: 107/1,000; exercise: 37/1,000 (4 to 334).
Seroconversion – H1N1 Acute	2 (70)	RR 1.30 (0.58 to 2.92)	Very low	Control: 226/1,000; exercise: 293/1,000 (130 to 659).
Seroconversion – H1N1 Chronic	1 (98)	RR 1.04 (0.66 to 1.62)	Low	Control: 431/1,000; exercise: 447/1,000 (286 to 699).
Seroconversion – H3N2 Acute	2 (71)	RR 0.84 (0.50 to 1.40)	Very low	Control: 469/1,000; exercise: 393/1,000 (236 to 654).
Seroconversion – H3N2 Chronic	1 (98)	RR 1.09 (0.81 to 1.45)	Low	Control: 627/1,000; exercise: 681/1,000 (510 to 908).
Seroconversion – Influenza B Acute	1 (45)	RR 0.24 (0.03 to 1.98)	Very low	Control: 182/1,000; exercise: 43/1,000 (5 to 359).
Seroconversion – Influenza B Chronic	1 (98)	RR 0.94 (0.60 to 1.48)	Low	Control: 451/1,000; exercise: 426/1,000 (271 to 667).
HAI titer – H1N1 Acute	1 (57)	MD 0.52 (-0.51 to 1.56)	Very low	Single-trial estimate; benefit and harm remain compatible.
HAI titer – H1N1 Chronic	2 (171)	MD 0.50 (-0.64 to 1.64)	Very low	Two trials; small-k sensitivity intervals are substantially wider.
HAI titer – H3N2 Acute	1 (57)	MD -0.47 (-1.38 to 0.44)	Very low	Single-trial estimate; benefit and harm remain compatible.
HAI titer – H3N2 Chronic	2 (171)	MD 0.27 (-0.17 to 0.72)	Very low	Two trials; small-k sensitivity intervals are substantially wider.
HAI titer – Influenza B Chronic	2 (171)	MD -0.14 (-0.64 to 0.36)	Very low	Two trials; small-k sensitivity intervals are substantially wider.

Certainty is assessed separately by outcome, antigen, and exposure. Randomized evidence starts at high certainty; nonrandomized evidence, reported separately in **Supplementary Table S6**, starts at low certainty. There is no automatic HAI-surrogate indirectness downgrade. Absolute binary risks use the aggregate observed control risk within that comparison multiplied by its RR; limits above 1,000 per 1,000 are capped at 1,000. These are illustrative immunogenicity probabilities, not infection risks. MDs are log_2_ units. Domain-specific reasons, including small-k imprecision and the chronic H1N1 inconsistency downgrade, appear in **Supplementary Table S6**.

## Discussion

This review assembled 22 reports but did not treat all exercise-related exposures as one intervention. The randomized analyses provide no clear evidence that acute exercise improves H1N1 or H3N2 seroprotection or seroconversion. Chronic-training titer estimates and the remaining single-trial comparisons are imprecise, and some are sensitive to interval method or study omission. These findings do not demonstrate equivalence or prove that exercise is ineffective. Observational and nonrandomized findings address different comparisons and remain insufficient to determine the causal effect of an exercise prescription.

### Comparison with previous work

The findings should be interpreted alongside, rather than as replacing, existing syntheses. Bohn-Goldbaum et al. examined individual-participant data from acute-exercise trials and separately considered habitual activity; their analysis did not establish an acute-exercise effect on seroconversion, although some activity associations favored greater response (35). Dinas et al. and Chastin et al. considered broader exercise, activity, or vaccination evidence and reported benefits in some analyses (36, 37). The influenza-specific Cochrane review also emphasized limitations in the available exercise evidence (38). Differences in study eligibility, exposure duration, assay, response metric, and availability of individual-level data can change a pooled result. They cannot, on their own, demonstrate that a positive result in another review was caused by confounding.

The observational findings suggest hypotheses rather than a consistent causal pattern. The activity gradient in Sadarangani (2022) was strongest for H3N2, while de Araujo (2015) and Keylock (2007) also reported antigen-specific associations in selected training or fitness groups (57, 66, 75). Frailty, comorbidity, sleep, diet, stress, and vaccination history may contribute to healthy-user differences, even after partial statistical adjustment (80). Measuring these covariates and distinguishing an observed exposure from a randomized intervention remain essential.

### Interpretation of the isolated signals

Source-reporting issues affect interpretation. The Hayney H1N1 fold-rise values cannot be reconciled as raw ratios with the same table’s seroconversion counts and were excluded (73). Elzayat’s two influenza B antigens were reported separately to avoid arbitrary selection into a generic B pool (74). Lee’s B/Brisbane fold-rise result favored exercise under the table-based reconstruction, but its uncertainty changed substantially when the discordant text-reported SDs were used (77). Together with a small sample and a reduced vaccine dose, this inconsistency prevents a robust inference of an antigen-specific benefit. Differences across titer, fold-rise, and threshold outcomes are not inherently contradictory because the metrics capture different features of the response.

### Why the trial evidence remains inconclusive

Several features may explain imprecise or heterogeneous findings. Pre-existing antibody and repeated vaccination can constrain the size of an additional response, particularly in older adults (4, 7). Reduced-dose vaccine studies examine a different antigenic challenge from routine full-dose vaccination and should not be generalized without qualification (63, 69, 77). Acute exercise and months of training have different proposed mechanisms, which is why they were separated rather than pooled because few trials were available. Small samples and incomplete numerical reporting limit precision (81). HAI is directly relevant to this review’s humoral question, but does not measure all components of immunity or prove prevention of clinical influenza; a particular increase in titer has no universally established patient-important meaning across settings (11, 12, 82).

Mechanistic plausibility remains distinct from evidence of an intervention effect. Acute exercise can alter immune-cell trafficking, and regular activity is associated with differences in the immune phenotype (19, 23, 28, 29). Studies of elite athletes and of primary responses to a novel antigen offer additional biological context, but do not supply the same adult randomized influenza-exercise contrast; the mixed-age athlete report was not included in the adult evidence set (83, 84). Reports of prolonged or intense exercise further suggest that duration and intensity may matter, without establishing the optimal exercise prescription around influenza vaccination (85, 86).

### Strengths and limitations

Strengths include separation of randomized intervention effects from observational associations, explicit distinction between acute and chronic exercise, assay- and antigen-specific outcome handling, and transparent reconstruction of log_2_ titer estimates. Numerical provenance distinguishes printed values, statistical transformations, digitized coordinates, and excluded results. Design-appropriate risk-of-bias domains are displayed without cross-tool aggregation, and small-k sensitivity intervals make uncertainty visible (49).

The evidence remains limited. Only five randomized reports contributed to a synthesis containing at least two trials, and several important comparisons are represented by one trial or no unambiguous numerical result. The maximum primary pool size is three; neither heterogeneity nor reporting bias can be characterized reliably. Some source tables contain internally inconsistent cells, and study-level means or variances are often not printed. Observational recruitment and cohort overlap further limit the independent information available. These problems cannot be solved by regarding an unreported number as a null response.

The review also has limitations. Trial registries and gray literature were not searched, and the humoral-outcome block may have missed reports in which relevant outcomes were not prominent in searchable fields. Original investigators were not contacted, so missing or inconsistent numerical data could not be clarified beyond the published reports and supplements. The search cutoff remained May 1, 2026. The exposure-specific stratification and additional sensitivity analyses were introduced during revision rather than prespecified. Neither a pooled dose–response curve nor a reliable age interaction could be estimated (51, 52). These limitations restrict confidence in completeness as well as precision.

### Implications for practice and research

The present evidence does not justify prescribing exercise specifically to improve influenza-vaccine antibody responses. Physical activity remains appropriate for its established cardiovascular, metabolic, musculoskeletal, and mental-health benefits (32, 34, 87). Conversely, these small immunogenicity studies do not establish vaccine-related harm from exercise or provide a definitive safety assessment. One trial reported fewer vaccine reactions after acute exercise, but this should not be generalized beyond the intervention and participants studied (60).

For research, the priorities follow from the limitations. Trials need to be substantially larger than those conducted so far, and should be powered for a prespecified, strain-specific antibody outcome rather than reporting antibody data as a secondary product of a trial designed for another purpose (81). Protocols and statistical analysis plans should be registered in advance, and every strain, threshold and timepoint reported, since concerns about selection of reported results were common, particularly in the randomized trials. Complete numerical reporting of group means, standard deviations and event counts, rather than figures alone, would immediately increase the amount of usable evidence. Exposure should be defined and quantified consistently, and separated by construct, so that acute exercise, structured training and habitual activity are not conflated; the framework for developing and evaluating complex interventions provides a useful structure for that work (88). Designs that reduce the ceiling effect, such as reduced-dose vaccine or antigens to which recipients have little prior immunity, are more likely to detect an adjuvant effect if one exists, and the introduction of vaccines against novel antigens has been identified as an opportunity to test behavioral determinants of vaccine response (89). Finally, because behavioral factors interact, future trials would gain from measuring sleep, stress and other concurrent behaviors alongside exercise, and from reporting them, since each of these has itself been linked to the antibody response to vaccination (90–92). Setting these priorities within an agreed research agenda for exercise immunology would help the field move beyond repeated small trials (93, 94).

## Conclusions

Low- or very-low-certainty evidence from a small number of randomized comparisons does not establish that acute exercise or chronic training improves the humoral response to influenza vaccination in adults. Exposure-specific estimates remain imprecise, and observational activity or fitness associations do not resolve the causal question. Larger trials, clearer allocation procedures, complete reporting of strain-specific numerical results, and reproducible review procedures are needed. No conclusion about prevention of clinical influenza follows directly from these HAI findings.

## Data Availability

The original contributions presented in the study are included in the article/**Supplementary Material**. Further inquiries can be directed to the corresponding author.
