# Supplementary material for: Exercise, physical activity, physical fitness, and humoral responses to influenza vaccination in adults: a systematic review and meta-analysis
Source: Front Immunol. 2026 Oct 8;17:1950491. doi: 10.3389/fimmu.2026.1950491 (PMC13686986; doi:10.3389/fimmu.2026.1950491)
Supplement: Supplementary file 1 [file Table1.docx]

**Supplementary Materials**

**Contents**

**Table S1. Database search strategy**

**Table S2. Outcome-level numerical data and analysis status**

**Table S3. Support for the risk-of-bias judgments**

**Table S4. Sensitivity analyses**

**Table S5. Nonpooled findings and observational exposure gradients**

**Table S6. GRADE evidence profile**

**Table S7. Numerical provenance and eligibility for quantitative synthesis**

**Figure S1. Individual HAI fold-rise estimates, without cross-exposure pooling**

**Figure S2. Within-study exposure gradients from the observational studies**

**Table S1. Database search strategy**

Six databases were searched from inception to May 1, 2026 using exercise/activity/fitness, influenza vaccination, and humoral-response concepts, without language or date limits. Trial registries and gray-literature sources were not searched. The database-specific search strings below are reproduced as executed, including alternative English spellings and database syntax. The humoral-outcome block may have reduced sensitivity when relevant outcomes were absent from searchable fields.

| **Database** | **Search string (as executed; cut-off 1 May 2026)** |
| --- | --- |
| #1 PubMed (MEDLINE) | (("Exercise"[Mesh] OR "Physical Fitness"[Mesh] OR "Physical Conditioning, Human"[Mesh] OR "Motor Activity"[Mesh] OR "Exercise Therapy"[Mesh] OR "Tai Ji"[Mesh] OR exercise[tiab] OR "physical activity"[tiab] OR "physical fitness"[tiab] OR "physical training"[tiab] OR aerobic[tiab] OR "resistance training"[tiab] OR "cardiorespiratory fitness"[tiab] OR "aerobic capacity"[tiab] OR VO₂max[tiab] OR walking[tiab] OR "tai chi"[tiab] OR taiji[tiab] OR qigong[tiab] OR exertion[tiab])) AND (("Influenza Vaccines"[Mesh] OR "Vaccination"[Mesh] OR "Immunization"[Mesh]) AND (influenza[tiab] OR flu[tiab]) OR "influenza vaccine*"[tiab] OR "flu vaccine*"[tiab] OR "influenza vaccination"[tiab] OR "influenza immuni*"[tiab]) AND (("Antibodies, Viral"[Mesh] OR "Hemagglutination Inhibition Tests"[Mesh] OR "Immunoglobulin G"[Mesh] OR "Immunity, Humoral"[Mesh] OR "Antibody Formation"[Mesh] OR antibod*[tiab] OR "hemagglutination inhibition"[tiab] OR "haemagglutination inhibition"[tiab] OR HAI[tiab] OR HI titer*[tiab] OR seroconversion[tiab] OR seroprotection[tiab] OR "immune response"[tiab] OR immunogenicity[tiab] OR "antibody titer*"[tiab] OR "antibody titre*"[tiab] OR immunoglobulin*[tiab] OR IgG[tiab] OR IgM[tiab] OR IgA[tiab] OR "fold rise"[tiab] OR "geometric mean titer*"[tiab])) |
| #2 Web of Science Core Collection | TS=((exercise OR "physical activity" OR "physical fitness" OR "physical training" OR aerobic OR "resistance training" OR "cardiorespiratory fitness" OR "aerobic capacity" OR VO₂max OR walking OR "tai chi" OR taiji OR qigong OR exertion) AND ("influenza vaccine*" OR "flu vaccine*" OR "influenza vaccination" OR "influenza immuni*" OR ((influenza OR flu) NEAR/3 (vaccin* OR immuni*))) AND (antibod* OR "hemagglutination inhibition" OR "haemagglutination inhibition" OR HAI OR seroconversion OR seroprotection OR immunogenicity OR "immune response" OR "antibody titer*" OR "antibody titre*" OR immunoglobulin* OR IgG OR IgM OR IgA OR "fold rise" OR "geometric mean tit*")) |
| #3 Embase | ('exercise'/exp OR 'physical activity'/exp OR 'fitness'/exp OR 'aerobic exercise'/exp OR 'resistance training'/exp OR 'tai chi'/exp OR 'qi gong'/exp OR exercise:ti,ab,kw OR 'physical activity':ti,ab,kw OR 'physical fitness':ti,ab,kw OR aerobic:ti,ab,kw OR 'resistance training':ti,ab,kw OR 'cardiorespiratory fitness':ti,ab,kw OR vo2max:ti,ab,kw OR walking:ti,ab,kw OR 'tai chi':ti,ab,kw OR taiji:ti,ab,kw OR qigong:ti,ab,kw OR exertion:ti,ab,kw) AND ('influenza vaccine'/exp OR 'influenza vaccination'/exp OR 'influenza vaccine*':ti,ab,kw OR 'flu vaccine*':ti,ab,kw OR 'influenza vaccination':ti,ab,kw OR 'influenza immuni*':ti,ab,kw) AND ('antibody'/exp OR 'hemagglutination inhibition test'/exp OR 'immunoglobulin g'/exp OR 'humoral immunity'/exp OR antibod*:ti,ab,kw OR 'hemagglutination inhibition':ti,ab,kw OR 'haemagglutination inhibition':ti,ab,kw OR hai:ti,ab,kw OR seroconversion:ti,ab,kw OR seroprotection:ti,ab,kw OR immunogenicity:ti,ab,kw OR 'antibody tit*':ti,ab,kw OR immunoglobulin*:ti,ab,kw OR igg:ti,ab,kw OR igm:ti,ab,kw OR iga:ti,ab,kw OR 'fold rise':ti,ab,kw OR 'geometric mean tit*':ti,ab,kw) |
| #4 Cochrane Library (CENTRAL) | ([mh Exercise] OR [mh "Physical Fitness"] OR [mh "Motor Activity"] OR [mh "Tai Ji"] OR exercise:ti,ab,kw OR "physical activity":ti,ab,kw OR "physical fitness":ti,ab,kw OR aerobic:ti,ab,kw OR "resistance training":ti,ab,kw OR "cardiorespiratory fitness":ti,ab,kw OR walking:ti,ab,kw OR "tai chi":ti,ab,kw OR qigong:ti,ab,kw) AND ([mh "Influenza Vaccines"] OR "influenza vaccine*":ti,ab,kw OR "flu vaccine*":ti,ab,kw OR "influenza vaccination":ti,ab,kw) AND ([mh "Hemagglutination Inhibition Tests"] OR [mh "Antibodies, Viral"] OR antibod*:ti,ab,kw OR "hemagglutination inhibition":ti,ab,kw OR HAI:ti,ab,kw OR seroconversion:ti,ab,kw OR seroprotection:ti,ab,kw OR immunogenicity:ti,ab,kw OR "antibody tit*":ti,ab,kw OR immunoglobulin*:ti,ab,kw OR IgG:ti,ab,kw) |
| #5 Scopus | TITLE-ABS-KEY((exercise OR "physical activity" OR "physical fitness" OR "physical training" OR aerobic OR "resistance training" OR "cardiorespiratory fitness" OR "aerobic capacity" OR VO₂max OR walking OR "tai chi" OR taiji OR qigong OR exertion) AND ("influenza vaccine*" OR "flu vaccine*" OR "influenza vaccination" OR "influenza immuni*") AND (antibod* OR "hemagglutination inhibition" OR "haemagglutination inhibition" OR HAI OR seroconversion OR seroprotection OR immunogenicity OR "immune response" OR "antibody titer*" OR "antibody titre*" OR immunoglobulin* OR IgG OR IgM OR IgA OR "fold rise" OR "geometric mean tit*")) |
| #6 SPORTDiscus (EBSCOhost) | (DE "EXERCISE" OR DE "PHYSICAL fitness" OR DE "AEROBIC exercises" OR DE "RESISTANCE training" OR DE "TAI chi" OR DE "QI gong" OR TI(exercise OR "physical activity" OR "physical fitness" OR aerobic OR "resistance training" OR "cardiorespiratory fitness" OR walking OR "tai chi" OR qigong) OR AB(exercise OR "physical activity" OR "physical fitness" OR aerobic OR "resistance training" OR "cardiorespiratory fitness" OR walking OR "tai chi" OR qigong)) AND (TI("influenza vaccin*" OR "flu vaccin*" OR "influenza immuni*") OR AB("influenza vaccin*" OR "flu vaccin*" OR "influenza immuni*")) AND (TI(antibod* OR "hemagglutination inhibition" OR HAI OR seroconversion OR seroprotection OR immunogenicity OR "antibody tit*" OR immunoglobulin* OR IgG OR IgM OR IgA) OR AB(antibod* OR "hemagglutination inhibition" OR HAI OR seroconversion OR seroprotection OR immunogenicity OR "antibody tit*" OR immunoglobulin* OR IgG OR IgM OR IgA)) |

AB, abstract; DE, descriptor (EBSCO); [mh], MeSH descriptor (Cochrane); [Mesh]/[tiab], MeSH term / title-abstract (PubMed); /exp, exploded Emtree term (Embase); :ti,ab,kw, title-abstract-keyword; NEAR/3 and NEAR/n, proximity operators; TI, title; TS, topic (Web of Science); TITLE-ABS-KEY, title-abstract-keyword (Scopus).

**Table S2. Outcome-level numerical data and analysis status**

| **Input ID / report** | **Outcome / exposure** | **Time** | **Metric** | **Exercise versus control** | **Effect (95% CI)** | **Analysis status** |
| --- | --- | --- | --- | --- | --- | --- |
| V2_001 Bohn-Goldbaum 2020 | Seroprotection – H1N1 Acute | 1 month | RR | 14/23 vs 15/21 | 0.85 (0.56 to 1.30) | Primary |
| V2_002 Bohn-Goldbaum 2020 | Seroprotection – H3N2 Acute | 1 month | RR | 21/22 vs 21/23 | 1.05 (0.89 to 1.22) | Primary |
| V2_003 Bohn-Goldbaum 2020 | Seroconversion – H1N1 Acute | 1 month | RR | 8/23 vs 6/21 | 1.22 (0.51 to 2.93) | Primary |
| V2_004 Bohn-Goldbaum 2020 | Seroconversion – H3N2 Acute | 1 month | RR | 12/23 vs 13/22 | 0.88 (0.52 to 1.49) | Primary |
| V2_005 Bohn-Goldbaum 2020 | Seroconversion – Influenza B Acute | 1 month | RR | 1/23 vs 4/22 | 0.24 (0.03 to 1.98) | Primary |
| V2_006 Elzayat 2021 | Seroprotection – H1N1 Acute | 6 weeks | RR | 9/16 vs 5/10 | 1.12 (0.53 to 2.39) | Primary |
| V2_007 Elzayat 2021 | Seroprotection – H3N2 Acute | 6 weeks | RR | 11/16 vs 4/10 | 1.72 (0.75 to 3.93) | Primary |
| V2_008 Elzayat 2021 | Seroconversion – H1N1 Acute | 6 weeks | RR | 3/16 vs 1/10 | 1.88 (0.22 to 15.63) | Primary |
| V2_009 Elzayat 2021 | Seroconversion – H3N2 Acute | 6 weeks | RR | 1/16 vs 2/10 | 0.31 (0.03 to 3.01) | Primary |
| V2_010 Elzayat 2021 | Seroprotection – B/Phuket/3073/2013 Acute | 6 weeks | RR | 2/16 vs 4/10 | 0.31 (0.07 to 1.40) | Separate B antigen |
| V2_011 Elzayat 2021 | Seroprotection – B/Colorado/06/2017 Acute | 6 weeks | RR | 0/16 vs 0/10 | Not estimable; double-zero | Separate B antigen |
| V2_012 Elzayat 2021 | Seroconversion – B/Phuket/3073/2013 Acute | 6 weeks | RR | 2/16 vs 1/10 | 1.25 (0.13 to 12.06) | Separate B antigen |
| V2_013 Elzayat 2021 | Seroconversion – B/Colorado/06/2017 Acute | 6 weeks | RR | 2/16 vs 0/10 | 3.24 (0.17 to 61.21) | Separate B antigen |
| V2_014 Ranadive 2014 | Seroprotection – H1N1 Acute | 4 weeks | RR | 11/27 vs 14/28 | 0.81 (0.45 to 1.46) | Primary |
| V2_015 Ranadive 2014 | Seroprotection – H3N2 Acute | 4 weeks | RR | 18/27 vs 20/28 | 0.93 (0.65 to 1.33) | Primary |
| V2_016 Ranadive 2014 | Seroprotection – Influenza B Acute | 4 weeks | RR | 1/27 vs 3/28 | 0.35 (0.04 to 3.12) | Primary |
| V2_017 Hayney 2014 | Seroprotection – H1N1 Chronic | 3 weeks | RR | 38/47 vs 43/51 | 0.96 (0.80 to 1.15) | Primary |
| V2_018 Hayney 2014 | Seroconversion – H1N1 Chronic | 3 weeks | RR | 21/47 vs 22/51 | 1.04 (0.66 to 1.62) | Primary |
| V2_019 Hayney 2014 | Seroconversion – H3N2 Chronic | 3 weeks | RR | 32/47 vs 32/51 | 1.09 (0.81 to 1.45) | Primary |
| V2_020 Hayney 2014 | Seroconversion – Influenza B Chronic | 3 weeks | RR | 20/47 vs 23/51 | 0.94 (0.60 to 1.48) | Primary |
| V2_021 Kohut 2004 | HAI titer – H1N1 Chronic | 4 weeks | MD (log₂) | 7.30 (SE 0.51) vs 6.08 (SE 0.34) | 1.22 (0.02 to 2.42) | Primary |
| V2_022 Kohut 2004 | HAI titer – H3N2 Chronic | 4 weeks | MD (log₂) | 6.73 (SE 0.22) vs 6.54 (SE 0.34) | 0.19 (-0.60 to 0.98) | Primary |
| V2_023 Kohut 2004 | HAI titer – Influenza B Chronic | 4 weeks | MD (log₂) | 5.74 (SE 0.32) vs 5.93 (SE 0.24) | -0.19 (-0.97 to 0.59) | Primary |
| V2_024 Woods 2009 | HAI titer – H1N1 Chronic | 3 weeks | MD (log₂) | 5.72 (SE 0.19) vs 5.69 (SE 0.19) | 0.03 (-0.50 to 0.56) | Primary |
| V2_025 Woods 2009 | HAI titer – H3N2 Chronic | 3 weeks | MD (log₂) | 6.55 (SE 0.19) vs 6.24 (SE 0.20) | 0.31 (-0.23 to 0.85) | Primary |
| V2_026 Woods 2009 | HAI titer – Influenza B Chronic | 3 weeks | MD (log₂) | 6.59 (SE 0.24) vs 6.69 (SE 0.23) | -0.10 (-0.75 to 0.55) | Primary |
| V2_027 Lee 2021 | HAI titer – H1N1 Acute | 4 weeks | MD (log₂) | n=38 vs 19; GMT/CI-derived | 0.52 (-0.51 to 1.56) | Primary |
| V2_028 Lee 2021 | HAI titer – H3N2 Acute | 4 weeks | MD (log₂) | n=38 vs 19; GMT/CI-derived | -0.47 (-1.38 to 0.44) | Primary |
| V2_029 Lee 2021 | HAI titer – B/Phuket/3073/2013 Acute | 4 weeks | MD (log₂) | n=38 vs 19; GMT/CI-derived | 0.21 (-0.43 to 0.85) | Separate B antigen |
| V2_030 Lee 2021 | HAI titer – B/Brisbane/60/2008 Acute | 4 weeks | MD (log₂) | n=38 vs 19; GMT/CI-derived | 0.56 (-0.04 to 1.15) | Separate B antigen |
| V2_031 Edwards 2010 | HAI titer – H1N1 Acute | 4 weeks | MD (log₂) | n=119 vs 39; GMT/CI-derived | 0.24 (-0.38 to 0.85) | Nonrandomized |
| V2_032 Edwards 2010 | HAI titer – H3N2 Acute | 4 weeks | MD (log₂) | n=119 vs 39; GMT/CI-derived | -0.09 (-0.90 to 0.71) | Nonrandomized |
| V2_033 Edwards 2010 | HAI titer – Influenza B Acute | 4 weeks | MD (log₂) | n=119 vs 39; GMT/CI-derived | -0.12 (-0.43 to 0.19) | Nonrandomized |
| V2_034 Bohn-Goldbaum 2020 | HAI titer – H1N1 Acute | 1 month | MD (log₂) | 1.58 (SD 0.647) vs 1.81 (printed SD 0.0653) | Not estimated; source ambiguity | Excluded; unresolved dispersion |
| V2_035 Bohn-Goldbaum 2020 | HAI titer – H3N2 Acute | 1 month | MD (log₂) | 1.91 (SD 0.556) vs 2.10 (SD 0.460) | -0.63 (-1.61 to 0.35) | Assumed log10 |
| V2_036 Bohn-Goldbaum 2020 | HAI titer – Influenza B Acute | 1 month | MD (log₂) | 1.70 (SD 0.484) vs 1.83 (SD 0.500) | -0.43 (-1.38 to 0.51) | Assumed log10 |
| V2_037 Lee 2021 | HAI fold rise – H1N1 Acute | 4 weeks | MD (fold) | 10.20 (SE 2.40) vs 10.80 (SE 8.52) | -0.60 (-17.95 to 16.75) | Supplementary; single trial |
| V2_038 Lee 2021 | HAI fold rise – H3N2 Acute | 4 weeks | MD (fold) | 11.00 (SE 4.08) vs 11.00 (SE 4.34) | 0.00 (-11.67 to 11.67) | Supplementary; single trial |
| V2_039 Hayney 2014 | HAI fold rise – H3N2 Chronic | 3 weeks | MD (fold) | 15.70 (SD 25.380) vs 10.54 (SD 19.410) | 5.16 (-3.84 to 14.16) | Supplementary; single trial |
| V2_040 Hayney 2014 | HAI fold rise – Influenza B Chronic | 3 weeks | MD (fold) | 6.84 (SD 13.140) vs 5.44 (SD 11.850) | 1.40 (-3.57 to 6.37) | Supplementary; single trial |
| V2_041 Hayney 2014 | Seroprotection – H3N2 Chronic | 3 weeks | RR | 45/47 vs 47/51 | 1.04 (0.94 to 1.15) | Ambiguous-cell sensitivity |
| V2_042 Hayney 2014 | Seroprotection – H3N2 Chronic | 3 weeks | RR | 47/47 vs 45/51 | 1.13 (1.02 to 1.26) | Ambiguous-cell sensitivity |
| V2_043 Lee 2021 | HAI fold rise – B/Phuket/3073/2013 Acute | 4 weeks | MD (fold) | 8.00 (SE 3.83) vs 3.00 (SE 1.02) | 5.00 (-2.76 to 12.76) | Supplementary; single trial |
| V2_044 Lee 2021 | HAI fold rise – B/Brisbane/60/2008 Acute | 4 weeks | MD (fold) | 7.60 (SE 2.32) vs 2.10 (SE 0.28) | 5.50 (0.92 to 10.08) | Supplementary; single trial |
| V2_045 Lee 2021 | HAI fold rise – B/Brisbane/60/2008 Acute | 4 weeks | MD (fold) | 7.60 (SD 12.900) vs 2.00 (SD 11.100) | 5.60 (-0.86 to 12.06) | Text-SD sensitivity |

Input IDs correspond to the machine-readable source data. Exercise arms are combined and the shared control is counted once. Primary estimates are randomized and exposure-specific. Both quadrivalent-vaccine B antigens are retained separately from generic B comparisons. V2_034 preserves the printed Bohn-Goldbaum H1N1 value without estimating an effect. Lee fold-rise estimates use approximate SEs from Table 3 confidence limits; V2_045 instead uses its discordant text-reported means and SDs and is not an additional trial. No primary binary comparison required correction. Elzayat B/Colorado seroconversion and the all-event exercise arm in one hypothetical Hayney H3N2 interpretation required 0.5 in all four cells; the double-zero Elzayat seroprotection comparison has no RR. Sources and reconstruction assumptions are detailed in Table S7. CI, confidence interval; GMT, geometric mean titer; MD, mean difference; RR, risk ratio; SD, standard deviation; SE, standard error.

**Table S3. Support for the risk-of-bias judgments**

| **Report** | **Tool** | **Overall humoral RoB** | **Result scope and support for judgment** |
| --- | --- | --- | --- |
| de Araujo et al. (2015) | ROBINS-E (2024) | High risk | Humoral result scope: HAI seroprotection - H1N1; HAI seroprotection - H3N2; HAI seroprotection - Influenza B. Training groups were self-selected and recruited from different community and sport settings. Important baseline differences included BMI, VO2max, activity dose, physical function, and prevaccination antibody levels, with no confounding-adjusted causal estimate. The two trained groups were pooled after separate comparisons were nonsignificant to increase power, creating serious selective-reporting concern. Native domains: D1:High; D2:Some concerns; D3:High; D4:Some concerns; D5:Low; D6:Low; D7:High. |
| Bachi et al. (2013) | ROBINS-E (2024) | High risk | Humoral result scope: Influenza-specific IgG; Influenza-specific IgM. Exercise and sedentary groups were non-randomized and recruited from different source populations; baseline BMI differed and no confounding-adjusted effect was reported. Table 1 states n=55 per group, but the sex counts sum to 45 per group, creating serious uncertainty about analyzed denominators. ELISA measurement was objective, but graph-only reporting and the absence of a prespecified analysis plan increase selective-reporting risk. Native domains: D1:High; D2:Some concerns; D3:High; D4:Some concerns; D5:High; D6:Low; D7:Some concerns. |
| Felismino et al. (2021) | ROBINS-E (2024) | High risk | Humoral result scope: Influenza-specific IgA; Influenza-specific IgG; Influenza-specific IgM. The training exposure was established before recruitment and was not allocated by investigators. Adiposity, habitual activity and sitting-time differences leave substantial residual confounding; all groups already exceeded 150 min/week of activity. Objective immunoglobulin measurement limits measurement bias, but does not resolve confounding. Cohort overlap with Monteiro 2020 is a synthesis-independence issue, not a separate bias domain. Native domains: D1:High; D2:Some concerns; D3:Some concerns; D4:Some concerns; D5:Low; D6:Low; D7:Some concerns. |
| Bohn-Goldbaum et al. (2020) | RoB 2 (2019) | Some concerns | Humoral result scope: HAI titer - H1N1; HAI titer - H3N2; HAI titer - Influenza B; HAI seroconversion - H1N1; HAI seroconversion - H3N2; HAI seroconversion - Influenza B; HAI seroprotection - H1N1; HAI seroprotection - H3N2; HAI seroprotection - Influenza B. Sex-stratified computer-generated randomization and sealed envelopes prepared by an independent researcher support the allocation process. One control participant withdrew after baseline. HAI outcomes were objectively measured and the trial was registered, but an accessible prespecified analysis plan for all strains, thresholds, and timepoints was not available. Native domains: D1:Low; D2:Low; D3:Low; D4:Low; D5:Some concerns. |
| Monteiro et al. (2020) | ROBINS-E (2024) | High risk | Humoral result scope: HAI fold change - H1N1. Exercise status was pre-existing, whereas glutamine/placebo supplementation was randomized. The eligible contrast compares combined-training/placebo with non-practicing/placebo participants and estimates an observational exercise-exposure association, not a randomized exercise effect. Baseline adiposity, activity and sitting-time differences were not adequately controlled. The reported HAI comparison was assessed with ROBINS-E; supplement randomization does not randomize exercise. Native domains: D1:High; D2:Some concerns; D3:Some concerns; D4:Some concerns; D5:Low; D6:Low; D7:Some concerns. |
| Woods et al. (2009) | RoB 2 (2019) | High risk | Humoral result scope: HAI titer - H1N1; HAI titer - H3N2; HAI titer - Influenza B; HAI seroprotection - H1N1; HAI seroprotection - H3N2; HAI seroprotection - Influenza B. Baseline-adaptive randomization and coded, blinded sample assessment support allocation and outcome measurement. However, 13 randomized participants never received their assigned intervention and three additional flexibility participants were excluded for HAI technical problems; the reported final 'intention-to-treat' analysis therefore omitted post-randomization participants. These deviations and missing outcomes could materially bias the effect. Native domains: D1:Low; D2:High; D3:High; D4:Low; D5:Some concerns. |
| Long et al. (2012) | RoB 2 (2019) | Some concerns | Humoral result scope: Composite HAI response; HAI titer - H1N1; HAI titer - H3N2; HAI titer - Influenza B. The CONSORT diagram reports 122 randomized participants, but sequence generation and allocation concealment were not described. One participant in each group was lost for documented reasons, leaving 120 analyzed. HAI measurement was objective, but no accessible prespecified analysis plan fixed the many age-stratum, vaccine, and strain analyses. Native domains: D1:Some concerns; D2:Low; D3:Low; D4:Low; D5:Some concerns. |
| Hallam et al. (2022) | RoB 2 (2019) | High risk | Humoral result scope: Influenza-specific IgG; Influenza-specific IgG - H1N1; Influenza-specific IgG - H3N2; Influenza-specific IgG fold change - H1N1; Influenza-specific IgG fold change - H3N2. Participants were described as randomly assigned, but sequence generation and concealment were not reported. In the monovalent H1N1 experiment, 3 of 19 randomized participants were excluded after receiving intranasal vaccine; one seasonal 45-minute participant was lost. ELISA measurement was objective, but no accessible registration or analysis plan constrained the many experiments, age strata, exercise durations, antigens, and timepoints. Multiple domain concerns materially reduce confidence for H1N1 results. Native domains: D1:Some concerns; D2:Low; D3:Some concerns; D4:Low; D5:Some concerns. |
| Campbell et al. (2010) | ROBINS-I (2016) | Serious risk | Humoral result scope: HAI titer - H1N1; HAI titer - H3N2; HAI titer - Influenza B. The report describes pseudo-random allocation balancing sex, without a demonstrably random concealed sequence. The review therefore treats the exercise contrast conservatively as nonrandomized. Residual confounding and selection at allocation are the main concerns; objective HAI measurement and nearly complete follow-up do not remove those concerns. Figure-only numerical reporting is a data-availability limitation, not by itself a reason for a high bias rating. Native domains: D1:Serious; D2:Moderate; D3:Low; D4:Low; D5:Low; D6:Low; D7:Moderate. |
| Keylock et al. (2007) | ROBINS-E (2024) | High risk | Result scope: H1N1 and influenza B seroconversion (high risk); continuous HAI-response analyses (some concerns). Pre-existing high- and low-fitness volunteers were recruited from community and sport settings. The seroconversion ratios use unadjusted event counts; groups differed in sex composition and adiposity, and vaccination history was self-reported. These important confounders were not controlled in the binary estimates. Covariate adjustment of separate continuous-response models does not remove this bias. Follow-up was complete and laboratory measurement was objective. Partially adjusted continuous-response outcomes retain some concerns; the report display uses the least favorable humoral-result judgment. Displayed native domains: D1:High; D2:Low; D3:Some concerns; D4:Low; D5:Low; D6:Low; D7:Some concerns. |
| Edwards et al. (2006) | RoB 2 (2019) | High risk | Humoral result scope: HAI titer - H3N2. Random allocation was stated, but sequence generation and concealment were not described. Occasional missing observations were acknowledged and HAI measurement was objective. The extracted H3N2 result is a sex-specific interaction selected from three strains and multiple condition-by-time analyses without an accessible prespecified analysis plan. Native domains: D1:Some concerns; D2:Low; D3:Some concerns; D4:Low; D5:High. |
| Edwards et al. (2007) | RoB 2 (2019) | Some concerns | Humoral result scope: HAI titer - H3N2; HAI titer - Influenza B. A pre-determined restricted random assignment was used, but allocation concealment was not described. Small amounts of missing follow-up data were present and laboratory immune outcomes were objectively measured. Sex interactions were analyzed, but no accessible protocol or statistical analysis plan fixed the complete set of result selections. Native domains: D1:Some concerns; D2:Low; D3:Some concerns; D4:Low; D5:Some concerns. |
| Edwards et al. (2010) | ROBINS-I (2016) | Serious risk | Humoral result scope: HAI titer - H1N1; HAI titer - H3N2; HAI titer - Influenza B. Allocation was described as pseudo-randomized while balancing sex; the sequence and concealment were not demonstrably random. The contrast is therefore treated as nonrandomized, with serious concern about confounding and moderate selection concern. Day-28 GMTs and confidence intervals are printed in Table 1 and are extractable; neither reduced vaccine dose nor table/figure format alone determines the bias judgment. Native domains: D1:Serious; D2:Moderate; D3:Low; D4:Low; D5:Low; D6:Low; D7:Moderate. |
| Ayling et al. (2018) | ROBINS-E (2024) | High risk | Humoral result scope: HAI seroprotection - H1N1; HAI seroprotection - H3N2; HAI seroprotection - Influenza B; Influenza-specific IgG - H1N1; Influenza-specific IgG - H3N2; Influenza-specific IgG - Influenza B. The extracted physical-activity associations are unadjusted correlations, leaving age, prior vaccination, baseline immunity, health, and correlated behaviors insufficiently controlled. Exposure was sampled on selected pedometer days; physical activity had 12.9% missing data without imputation. Analyses were exploratory and not multiplicity-corrected, although immune outcomes were objectively measured. Native domains: D1:High; D2:Some concerns; D3:Some concerns; D4:Low; D5:Some concerns; D6:Low; D7:Some concerns. |
| Kohut et al. (2002) | ROBINS-E (2024) | High risk | Humoral result scope: Influenza-specific IgG; Influenza-specific IgM. Activity categories described behavior in the preceding year rather than an assigned intervention. Health and lifestyle confounding was insufficiently controlled, and no prevaccination antibody baseline was available. Objective ELISA measurements were obtained after vaccination. The humoral-risk rating is separate from the greater measurement concern for participant-reported influenza-like illness. Native domains: D1:High; D2:Some concerns; D3:Some concerns; D4:Some concerns; D5:Low; D6:Low; D7:Some concerns. |
| Kohut et al. (2004) | RoB 2 (2019) | Some concerns | Humoral result scope: HAI fold change - H1N1; HAI fold change - H3N2; HAI fold change - Influenza B; HAI titer - H1N1; HAI titer - H3N2; HAI titer - Influenza B. The full text states that participants were randomly assigned, but sequence generation and allocation concealment were not described. One control participant was removed after a cancer diagnosis; this isolated, outcome-independent loss is unlikely to materially bias results. HAI was objectively measured with each participant's timepoints run on the same plate, but no accessible prespecified analysis plan was identified. Native domains: D1:Some concerns; D2:Low; D3:Low; D4:Low; D5:Some concerns. |
| Hayney et al. (2014) | RoB 2 (2019) | Some concerns | Humoral result scope: HAI fold change - H1N1; HAI fold change - H3N2; HAI fold change - Influenza B; HAI seroconversion - H1N1; HAI seroconversion - H3N2; HAI seroconversion - Influenza B; HAI seroprotection - H1N1; HAI seroprotection - H3N2. Random assignment to exercise, MBSR, or waitlist was reported, but sequence generation and concealment were not described in this report. Attrition was limited (149 of 154 completed), and HAI outcomes were objective. No accessible analysis plan fixed all antibody metrics, and several published seroprotection cells have denominator or column inconsistencies. Native domains: D1:Some concerns; D2:Low; D3:Low; D4:Low; D5:Some concerns. |
| Elzayat et al. (2021) | RoB 2 (2019) | Some concerns | Humoral result scope: HAI fold change - H1N1; HAI fold change - H3N2; HAI fold change - B/Colorado/06/2017; HAI fold change - B/Phuket/3073/2013; HAI seroconversion - H1N1; HAI seroconversion - H3N2; HAI seroconversion - B/Colorado/06/2017; HAI seroconversion - B/Phuket/3073/2013; HAI seroprotection - H1N1; HAI seroprotection - H3N2; HAI seroprotection - B/Colorado/06/2017; HAI seroprotection - B/Phuket/3073/2013. The random sequence was generated in Excel, but allocation concealment was not reported. Follow-up was substantially complete. HAI assays were performed by intervention-blinded researchers in duplicate with independent data verification, and the registered trial reported the planned fold-rise, seroprotection, and seroconversion outcomes. Native domains: D1:Some concerns; D2:Low; D3:Low; D4:Low; D5:Low. |
| Sadarangani et al. (2022) | ROBINS-E (2024) | High risk | Humoral result scope: Any-strain HAI seroconversion; HAI fold rise - H1N1; HAI fold rise - H3N2; HAI fold rise - Influenza B; HAI seroconversion - H1N1; HAI seroconversion - H3N2; HAI seroconversion - Influenza B. The multivariable models adjusted many measured characteristics, including baseline HAI and prior vaccination, but important residual health-behavior and frailty confounding remains. Physical activity was assessed by interviewer-administered RAPA rather than direct monitoring and the authors acknowledge recall and social-desirability bias. Retention was high and HAI outcomes were objectively measured; extensive predictor testing raises some selective-analysis concern. Native domains: D1:High; D2:Some concerns; D3:Low; D4:Low; D5:Low; D6:Low; D7:Some concerns. |
| Ranadive et al. (2014) | RoB 2 (2019) | Some concerns | Humoral result scope: Composite HAI response; HAI seroprotection - H1N1; HAI seroprotection - H3N2; HAI seroprotection - Influenza B. Participants were randomized to exercise or no exercise, but sequence generation and concealment were not described. Four of 59 participants dropped after the initial visit for documented reasons. Participants and researchers were blinded to vaccine versus sham timing, HAI was objective, and the sex analysis was stated as an aim; however no accessible prespecified analysis plan was identified. Native domains: D1:Some concerns; D2:Low; D3:Low; D4:Low; D5:Some concerns. |
| Lee et al. (2021) | RoB 2 (2019) | Some concerns | Humoral result scope: HAI fold change - H1N1; HAI fold change - H3N2; HAI fold change - Influenza B/Victoria; HAI fold change - Influenza B/Yamagata; HAI titer - H1N1; HAI titer - H3N2; HAI titer - Influenza B/Victoria; HAI titer - Influenza B/Yamagata. A computer-generated sequence was used and allocation was concealed from staff and participants until clinic attendance. The relevant control and pre-arm groups each contained 19 participants, whereas the pre-leg allocation initially contained 20 but 19 were retained in the current analysis without a clear reason. HAI was performed by a WHO reference laboratory; no accessible analysis plan fixed pooled versus arm-specific comparisons. Native domains: D1:Low; D2:Low; D3:Some concerns; D4:Low; D5:Some concerns. |
| Yang et al. (2007) | ROBINS-I (2016) | Serious risk | Humoral result scope: Composite HAI response; HAI seroprotection - H1N1; HAI seroprotection - H3N2; HAI seroprotection - Influenza B. The authors state that this was not a true randomized controlled trial: only 14 controls came from the randomized parent trial and nine additional controls were recruited nonrandomly. Group differences in age, recruitment and attention can confound the exercise effect. Blinded antibody measurement and longitudinal handling of a missing timepoint do not resolve those problems. Native domains: D1:Serious; D2:Serious; D3:Low; D4:Moderate; D5:Low; D6:Low; D7:Serious. |

The scope is humoral vaccine-response results. The overall rating is the least favorable relevant outcome judgment; clinical illness and cell-mediated outcomes do not determine the displayed humoral rating. The outcome-level assessment file provides the separate records. RoB 2 domains: randomization, deviations, missing data, outcome measurement, selection of the reported result. ROBINS-I (2016): confounding, selection of participants, classification of intervention, deviations, missing data, measurement, selection of the reported result. ROBINS-E (2024): confounding, exposure measurement, selection, post-exposure interventions, missing data, measurement, selection of the reported result. Domain numbers across tools are not interchangeable.

**Table S4. Sensitivity analyses**

| **Outcome / exposure** | **Sensitivity analysis** | **k** | **Effect (95% CI)** | **I²** | **Interval / contributing report(s)** |
| --- | --- | --- | --- | --- | --- |
| Seroprotection – H1N1 Acute | Modified Knapp-Hartung, scale bounded below by 1 | 3 | 0.88 (0.44 to 1.75) | 0% | Modified Knapp-Hartung; Bohn-Goldbaum 2020; Elzayat 2021; Ranadive 2014 |
| Seroprotection – H1N1 Acute | Omit Bohn-Goldbaum 2020 | 2 | 0.92 (0.58 to 1.46) | 0% | Normal; Elzayat 2021; Ranadive 2014 |
| Seroprotection – H1N1 Acute | Omit Elzayat 2021 | 2 | 0.84 (0.59 to 1.18) | 0% | Normal; Bohn-Goldbaum 2020; Ranadive 2014 |
| Seroprotection – H1N1 Acute | Omit Ranadive 2014 | 2 | 0.91 (0.63 to 1.32) | 0% | Normal; Bohn-Goldbaum 2020; Elzayat 2021 |
| Seroprotection – H1N1 Acute | Bohn-Goldbaum nominal 23/23 denominators | 3 | 0.93 (0.67 to 1.28) | 0% | Normal; Bohn-Goldbaum 2020; Elzayat 2021; Ranadive 2014 |
| Seroprotection – H3N2 Acute | Modified Knapp-Hartung, scale bounded below by 1 | 3 | 1.04 (0.77 to 1.42) | 0% | Modified Knapp-Hartung; Bohn-Goldbaum 2020; Elzayat 2021; Ranadive 2014 |
| Seroprotection – H3N2 Acute | Omit Bohn-Goldbaum 2020 | 2 | 1.12 (0.65 to 1.95) | 43% | Normal; Elzayat 2021; Ranadive 2014 |
| Seroprotection – H3N2 Acute | Omit Elzayat 2021 | 2 | 1.03 (0.89 to 1.18) | 0% | Normal; Bohn-Goldbaum 2020; Ranadive 2014 |
| Seroprotection – H3N2 Acute | Omit Ranadive 2014 | 2 | 1.13 (0.79 to 1.60) | 25% | Normal; Bohn-Goldbaum 2020; Elzayat 2021 |
| Seroprotection – H3N2 Acute | Bohn-Goldbaum nominal 23/23 denominators | 3 | 1.01 (0.86 to 1.18) | 0% | Normal; Bohn-Goldbaum 2020; Elzayat 2021; Ranadive 2014 |
| Seroconversion – H1N1 Acute | Modified Knapp-Hartung, scale bounded below by 1 | 2 | 1.30 (0.01 to 249.08) | 0% | Modified Knapp-Hartung; Bohn-Goldbaum 2020; Elzayat 2021 |
| Seroconversion – H1N1 Acute | Omit Bohn-Goldbaum 2020 | 1 | 1.88 (0.22 to 15.63) | — | Normal; Elzayat 2021 |
| Seroconversion – H1N1 Acute | Omit Elzayat 2021 | 1 | 1.22 (0.51 to 2.93) | — | Normal; Bohn-Goldbaum 2020 |
| Seroconversion – H1N1 Acute | Bohn-Goldbaum nominal 23/23 denominators | 2 | 1.40 (0.62 to 3.18) | 0% | Normal; Bohn-Goldbaum 2020; Elzayat 2021 |
| Seroconversion – H3N2 Acute | Modified Knapp-Hartung, scale bounded below by 1 | 2 | 0.84 (0.03 to 22.86) | 0% | Modified Knapp-Hartung; Bohn-Goldbaum 2020; Elzayat 2021 |
| Seroconversion – H3N2 Acute | Omit Bohn-Goldbaum 2020 | 1 | 0.31 (0.03 to 3.01) | — | Normal; Elzayat 2021 |
| Seroconversion – H3N2 Acute | Omit Elzayat 2021 | 1 | 0.88 (0.52 to 1.49) | — | Normal; Bohn-Goldbaum 2020 |
| Seroconversion – H3N2 Acute | Bohn-Goldbaum nominal 23/23 denominators | 2 | 0.87 (0.52 to 1.46) | 0% | Normal; Bohn-Goldbaum 2020; Elzayat 2021 |
| Seroconversion – Influenza B Acute | Bohn-Goldbaum nominal 23/23 denominators | 1 | 0.25 (0.03 to 2.07) | — | Normal; Bohn-Goldbaum 2020 |
| HAI titer – H1N1 Chronic | Modified Knapp-Hartung, scale bounded below by 1 | 2 | 0.50 (-6.89 to 7.88) | 68% | Modified Knapp-Hartung; Kohut 2004; Woods 2009 |
| HAI titer – H1N1 Chronic | Omit Kohut 2004 | 1 | 0.03 (-0.50 to 0.56) | — | Normal; Woods 2009 |
| HAI titer – H1N1 Chronic | Omit Woods 2009 | 1 | 1.22 (0.02 to 2.42) | — | Normal; Kohut 2004 |
| HAI titer – H3N2 Chronic | Modified Knapp-Hartung, scale bounded below by 1 | 2 | 0.27 (-2.62 to 3.17) | 0% | Modified Knapp-Hartung; Kohut 2004; Woods 2009 |
| HAI titer – H3N2 Chronic | Omit Kohut 2004 | 1 | 0.31 (-0.23 to 0.85) | — | Normal; Woods 2009 |
| HAI titer – H3N2 Chronic | Omit Woods 2009 | 1 | 0.19 (-0.60 to 0.98) | — | Normal; Kohut 2004 |
| HAI titer – Influenza B Chronic | Modified Knapp-Hartung, scale bounded below by 1 | 2 | -0.14 (-3.39 to 3.11) | 0% | Modified Knapp-Hartung; Kohut 2004; Woods 2009 |
| HAI titer – Influenza B Chronic | Omit Kohut 2004 | 1 | -0.10 (-0.75 to 0.55) | — | Normal; Woods 2009 |
| HAI titer – Influenza B Chronic | Omit Woods 2009 | 1 | -0.19 (-0.97 to 0.59) | — | Normal; Kohut 2004 |
| HAI titer – H1N1 Acute | Add pseudo-randomized Edwards 2010 (mixed-design sensitivity only) | 2 | 0.31 (-0.22 to 0.84) | 0% | Normal; Lee 2021; Edwards 2010 |
| HAI titer – H3N2 Acute | Assume Bohn-Goldbaum base-10 logarithms | 2 | -0.54 (-1.21 to 0.12) | 0% | Normal; Lee 2021; Bohn-Goldbaum 2020 |
| HAI titer – H3N2 Acute | Assumed-log10 plus modified Knapp-Hartung | 2 | -0.54 (-4.88 to 3.79) | 0% | Modified Knapp-Hartung; Lee 2021; Bohn-Goldbaum 2020 |
| HAI titer – H3N2 Acute | Add pseudo-randomized Edwards 2010 (mixed-design sensitivity only) | 2 | -0.26 (-0.86 to 0.35) | 0% | Normal; Lee 2021; Edwards 2010 |
| HAI titer – Influenza B Acute | Assume Bohn-Goldbaum base-10 logarithms | 1 | -0.43 (-1.38 to 0.51) | — | Normal; Bohn-Goldbaum 2020 |
| Seroprotection – H3N2 Chronic | Whole cells reassigned to match arm Ns | 1 | 1.04 (0.94 to 1.15) | — | Normal; Hayney 2014 |
| Seroprotection – H3N2 Chronic | Numerators retained under printed column labels | 1 | 1.13 (1.02 to 1.26) | — | Normal; Hayney 2014 |
| HAI fold rise – B/Brisbane/60/2008 Acute | Lee B/Brisbane: Results-text means and SDs rather than Table 3 confidence limits | 1 | 5.60 (-0.86 to 12.06) | — | Normal; Lee 2021 |

These are revision-stage robustness checks, not prespecified confirmatory tests. A remaining single trial is labeled k = 1 and is not a meta-analysis. Bounded modified Knapp–Hartung uses max(1, residual scale) and a t distribution with k − 1 degrees of freedom; primary intervals use a normal approximation. Assumed-log10 Bohn-Goldbaum analyses are restricted to H3N2 and influenza B; H1N1 is excluded because its dispersion and logarithm base remain unresolved. Alternative Hayney cell interpretations and the Lee B/Brisbane text-SD calculation describe source ambiguity rather than confirmed corrections. The Edwards sensitivity mixes design classes only as a robustness check and does not alter the randomized primary estimates. Hallam figure-derived readings and ambiguous Hayney H1N1 fold-rise values are excluded.

**Table S5. Nonpooled findings and observational exposure gradients**

| **Report / analysis** | **Contrast** | **Outcome** | **Exposure level** | **Events/N or k** | **Effect (95% CI)** | **Interpretation** |
| --- | --- | --- | --- | --- | --- | --- |
| de Araujo et al. (2015) | Training lifestyle vs never trained | Seroprotection H1N1 (6 wk) | Moderately trained | 17/23 | RR 1.11 (0.72 to 1.71) | ROBINS-E high; single study |
| de Araujo et al. (2015) | Training lifestyle vs never trained | Seroprotection H1N1 (6 wk) | Intensely trained | 16/22 | RR 1.09 (0.70 to 1.69) | ROBINS-E high; single study |
| de Araujo et al. (2015) | Training lifestyle vs never trained | Seroprotection H3N2 (6 wk) | Moderately trained | 19/23 | RR 1.38 (0.87 to 2.17) | ROBINS-E high; single study |
| de Araujo et al. (2015) | Training lifestyle vs never trained | Seroprotection H3N2 (6 wk) | Intensely trained | 20/22 | RR 1.52 (0.98 to 2.34) | ROBINS-E high; single study |
| de Araujo et al. (2015) | Training lifestyle vs never trained | Seroprotection Influenza B (6 wk) | Moderately trained | 14/23 | RR 1.83 (0.83 to 4.01) | ROBINS-E high; single study |
| de Araujo et al. (2015) | Training lifestyle vs never trained | Seroprotection Influenza B (6 wk) | Intensely trained | 15/22 | RR 2.05 (0.95 to 4.42) | ROBINS-E high; single study |
| Sadarangani et al. (2022) | RAPA category vs rare | HAI fold rise H1N1 (~4 wk) | Light | — | Ratio 0.97 (0.44 to 2.11) | ROBINS-E high; adjusted |
| Sadarangani et al. (2022) | RAPA category vs rare | HAI fold rise H1N1 (~4 wk) | Intermediate | — | Ratio 0.67 (0.26 to 1.72) | ROBINS-E high; adjusted |
| Sadarangani et al. (2022) | RAPA category vs rare | HAI fold rise H1N1 (~4 wk) | Moderate | — | Ratio 0.60 (0.26 to 1.35) | ROBINS-E high; adjusted |
| Sadarangani et al. (2022) | RAPA category vs rare | HAI fold rise H1N1 (~4 wk) | Vigorous | — | Ratio 0.81 (0.29 to 2.28) | ROBINS-E high; adjusted |
| Sadarangani et al. (2022) | RAPA category vs rare | HAI fold rise H3N2 (~4 wk) | Light | — | Ratio 2.04 (1.06 to 3.91) | ROBINS-E high; adjusted; trend p=0.015 |
| Sadarangani et al. (2022) | RAPA category vs rare | HAI fold rise H3N2 (~4 wk) | Intermediate | — | Ratio 2.52 (1.14 to 5.57) | ROBINS-E high; adjusted; trend p=0.015 |
| Sadarangani et al. (2022) | RAPA category vs rare | HAI fold rise H3N2 (~4 wk) | Moderate | — | Ratio 2.78 (1.41 to 5.51) | ROBINS-E high; adjusted; trend p=0.015 |
| Sadarangani et al. (2022) | RAPA category vs rare | HAI fold rise H3N2 (~4 wk) | Vigorous | — | Ratio 2.53 (1.07 to 5.99) | ROBINS-E high; adjusted; trend p=0.015 |
| Sadarangani et al. (2022) | RAPA category vs rare | HAI fold rise Influenza B (~4 wk) | Light | — | Ratio 0.97 (0.66 to 1.43) | ROBINS-E high; adjusted |
| Sadarangani et al. (2022) | RAPA category vs rare | HAI fold rise Influenza B (~4 wk) | Intermediate | — | Ratio 1.14 (0.71 to 1.82) | ROBINS-E high; adjusted |
| Sadarangani et al. (2022) | RAPA category vs rare | HAI fold rise Influenza B (~4 wk) | Moderate | — | Ratio 0.92 (0.61 to 1.38) | ROBINS-E high; adjusted |
| Sadarangani et al. (2022) | RAPA category vs rare | HAI fold rise Influenza B (~4 wk) | Vigorous | — | Ratio 0.60 (0.36 to 1.00) | ROBINS-E high; adjusted |
| Keylock et al. (2007) | High vs low fitness | Seroconversion H1N1 (6 wk) | High fitness | 8/13 vs 3/13 | RR 2.67 (0.90 to 7.86) | ROBINS-E high; unadjusted single-study ratio |
| Keylock et al. (2007) | High vs low fitness | Seroconversion Influenza B (6 wk) | High fitness | 7/13 vs 2/13 | RR 3.50 (0.89 to 13.78) | ROBINS-E high; unadjusted single-study ratio |
| Kohut et al. (2002) | Moderately active vs sedentary | Influenza-like illness | Moderately active | 3/25 vs 1/15 | RR 1.80 (0.21 to 15.78) | ROBINS-E high; sparse events |
| Hallam 2022 | 90-min postvaccination exercise vs rest | ELISA antibody fold change | — | — | Not pooled | Reported greater responses after 90-min exercise; no HAI pool; exploratory figure readings excluded. |
| Yang 2007 | Taiji/Qigong vs mixed-source controls | Composite HAI response | — | — | Not pooled | Nonrandomized comparison; strain-pooled modeling and baseline differences; no randomized HAI estimate. |
| Bachi 2013 | Observed regular exercise vs sedentary lifestyle | Influenza-specific IgG/IgM | — | — | Not pooled | Higher postvaccination antibody levels reported; unadjusted exposure comparison and inconsistent sample counts. |
| Felismino 2021 | Observed combined-training vs non-practicing group | Influenza-specific IgA/IgG/IgM | — | — | Not pooled | Selected within-group antibody changes; not a confounding-adjusted exercise effect; overlaps Monteiro cohort. |
| Monteiro 2020 | Training/placebo vs non-practicing/placebo | HAI and influenza-specific antibody | — | — | Not pooled | Supplement randomization does not randomize exercise exposure; exercise-placebo contrast considered separately. |
| Ayling 2018 | Habitual steps as observed exposure | HAI and strain-specific IgG | — | — | Not pooled | Exploratory correlations; exact outcome-specific N not consistently printed; no conversion of p values into arm-level effects. |
| Campbell 2010 | Pseudo-randomized exercise timing | HAI titers | — | — | Not pooled | Group-specific plotted values lack a complete compatible numerical summary; narrative only. |
| Edwards 2006 / 2007 | Acute exercise vs rest | HAI titers | — | — | Not pooled | Sex-/strain-specific findings and figure-based reporting; complete arm-level means and dispersion unavailable. |
| Long 2012 | Acute walking vs rest, by age | HAI response | — | — | Not pooled | Mixed-age strata and responder/graph reporting did not provide a complete compatible arm-level titer estimate. |
| Age subgroup | Within-exposure age comparison | HAI response | — | — | Not pooled | No reliable age interaction: too few comparable randomized trials after separating acute/chronic exposure and design. |

Observed exposure groups are not randomized interventions. de Araujo contrasts use the source-specific six-week denominators, including 15 never-trained participants with outcome data; the larger enrollment count is not substituted. Sadarangani ratios are multivariable-adjusted; de Araujo and the displayed Keylock event-count ratios are unadjusted. The Kohut (2002) illness comparison is moderately active versus sedentary, not vigorous activity, and is not a humoral endpoint. No pooled dose–response curve or age interaction is fitted. Age, exposure duration, and design are too closely aligned in the sparse trial evidence to support the former cross-exposure age pools.

**Table S6. GRADE evidence profile**

| **Outcome / exposure** | **Design / starting level** | **k / N** | **Effect (95% CI)** | **Final GRADE** | **Domains and reasons** |
| --- | --- | --- | --- | --- | --- |
| Seroprotection – H1N1 Acute | Randomized; starts high | 3 / 125 | 0.88 (0.65 to 1.21) | Low | RoB -1; inconsistency 0; indirectness 0; imprecision -1; publication bias: not assessable. Risk of bias −1: allocation/report-selection concerns in the contributing randomized trial(s). Inconsistency: no additional downgrade; sparse evidence limits interpretation of I². Imprecision −1: The information size is limited and the interval does not establish an immunogenicity benefit or equivalence; small-k interval sensitivity further reduces precision. Indirectness 0: HAI is a direct measure for the humoral-immunogenicity question; acute and chronic evidence is separated. No inference about prevention of clinical influenza is made. Publication bias: cannot be assessed statistically (k<10); not assumed absent and not automatically downgraded. |
| Seroprotection – H3N2 Acute | Randomized; starts high | 3 / 126 | 1.04 (0.91 to 1.20) | Low | RoB -1; inconsistency 0; indirectness 0; imprecision -1; publication bias: not assessable. Risk of bias −1: allocation/report-selection concerns in the contributing randomized trial(s). Inconsistency: no additional downgrade; sparse evidence limits interpretation of I². Imprecision −1: The information size is limited and the interval does not establish an immunogenicity benefit or equivalence; small-k interval sensitivity further reduces precision. Indirectness 0: HAI is a direct measure for the humoral-immunogenicity question; acute and chronic evidence is separated. No inference about prevention of clinical influenza is made. Publication bias: cannot be assessed statistically (k<10); not assumed absent and not automatically downgraded. |
| Seroconversion – H1N1 Acute | Randomized; starts high | 2 / 70 | 1.30 (0.58 to 2.92) | Very low | RoB -1; inconsistency 0; indirectness 0; imprecision -2; publication bias: not assessable. Risk of bias −1: allocation/report-selection concerns in the contributing randomized trial(s). Inconsistency: no additional downgrade; sparse evidence limits interpretation of I². Imprecision −2: Only two small trials, with few events, yield intervals compatible with substantial decreases and increases. Indirectness 0: HAI is a direct measure for the humoral-immunogenicity question; acute and chronic evidence is separated. No inference about prevention of clinical influenza is made. Publication bias: cannot be assessed statistically (k<10); not assumed absent and not automatically downgraded. |
| Seroconversion – H3N2 Acute | Randomized; starts high | 2 / 71 | 0.84 (0.50 to 1.40) | Very low | RoB -1; inconsistency 0; indirectness 0; imprecision -2; publication bias: not assessable. Risk of bias −1: allocation/report-selection concerns in the contributing randomized trial(s). Inconsistency: no additional downgrade; sparse evidence limits interpretation of I². Imprecision −2: Only two small trials, with few events, yield intervals compatible with substantial decreases and increases. Indirectness 0: HAI is a direct measure for the humoral-immunogenicity question; acute and chronic evidence is separated. No inference about prevention of clinical influenza is made. Publication bias: cannot be assessed statistically (k<10); not assumed absent and not automatically downgraded. |
| Seroconversion – Influenza B Acute | Randomized; starts high | 1 / 45 | 0.24 (0.03 to 1.98) | Very low | RoB -1; inconsistency 0; indirectness 0; imprecision -2; publication bias: not assessable. Risk of bias −1: allocation/report-selection concerns in the contributing randomized trial(s). Inconsistency: not assessable in a single trial; no automatic downgrade. Imprecision −2: A single small trial and a wide confidence interval permit markedly different conclusions about immunogenicity. Indirectness 0: HAI is a direct measure for the humoral-immunogenicity question; acute and chronic evidence is separated. No inference about prevention of clinical influenza is made. Publication bias: cannot be assessed statistically (k<10); not assumed absent and not automatically downgraded. |
| Seroprotection – Influenza B Acute | Randomized; starts high | 1 / 55 | 0.35 (0.04 to 3.12) | Very low | RoB -1; inconsistency 0; indirectness 0; imprecision -2; publication bias: not assessable. Risk of bias −1: allocation/report-selection concerns in the contributing randomized trial(s). Inconsistency: not assessable in a single trial; no automatic downgrade. Imprecision −2: A single small trial and a wide confidence interval permit markedly different conclusions about immunogenicity. Indirectness 0: HAI is a direct measure for the humoral-immunogenicity question; acute and chronic evidence is separated. No inference about prevention of clinical influenza is made. Publication bias: cannot be assessed statistically (k<10); not assumed absent and not automatically downgraded. |
| Seroprotection – H1N1 Chronic | Randomized; starts high | 1 / 98 | 0.96 (0.80 to 1.15) | Low | RoB -1; inconsistency 0; indirectness 0; imprecision -1; publication bias: not assessable. Risk of bias −1: incompletely reported allocation and result-selection procedures. Inconsistency: not assessable in one trial. Indirectness: no downgrade for this humoral outcome. Imprecision −1: A single trial includes 98 participants, with 38/47 versus 43/51 protected. The interval permits a meaningful decrease as well as little or some increase in response; the information size is limited. Publication bias cannot be assessed reliably; no additional downgrade was applied. |
| Seroconversion – H1N1 Chronic | Randomized; starts high | 1 / 98 | 1.04 (0.66 to 1.62) | Low | RoB -1; inconsistency 0; indirectness 0; imprecision -1; publication bias: not assessable. Risk of bias −1: incompletely reported allocation and result-selection procedures. Inconsistency: not assessable in one trial. Indirectness: no downgrade for this humoral outcome. Imprecision −1: A single trial includes 98 participants and 43 seroconversions. Its interval permits appreciable decreases and increases in response. Publication bias cannot be assessed reliably; no additional downgrade was applied. |
| Seroconversion – H3N2 Chronic | Randomized; starts high | 1 / 98 | 1.09 (0.81 to 1.45) | Low | RoB -1; inconsistency 0; indirectness 0; imprecision -1; publication bias: not assessable. Risk of bias −1: incompletely reported allocation and result-selection procedures. Inconsistency: not assessable in one trial. Indirectness: no downgrade for this humoral outcome. Imprecision −1: A single trial includes 98 participants and 64 seroconversions. Its interval permits reduced response and a potentially important increase. Publication bias cannot be assessed reliably; no additional downgrade was applied. |
| Seroconversion – Influenza B Chronic | Randomized; starts high | 1 / 98 | 0.94 (0.60 to 1.48) | Low | RoB -1; inconsistency 0; indirectness 0; imprecision -1; publication bias: not assessable. Risk of bias −1: incompletely reported allocation and result-selection procedures. Inconsistency: not assessable in one trial. Indirectness: no downgrade for this humoral outcome. Imprecision −1: A single trial includes 98 participants and 43 seroconversions. Its interval permits appreciable decreases and increases in response. Publication bias cannot be assessed reliably; no additional downgrade was applied. |
| HAI titer – H1N1 Chronic | Randomized; starts high | 2 / 171 | 0.50 (-0.64 to 1.64) | Very low | RoB -1; inconsistency -1; indirectness 0; imprecision -2; publication bias: not assessable. Risk of bias −1: allocation/report-selection concerns in the contributing randomized trial(s); Woods 2009 also excludes randomized participants from its analysis. Inconsistency −1: the two chronic-training H1N1 estimates differ (I² approximately 68%); only two trials make the magnitude of heterogeneity uncertain. Imprecision −2: Two trials (171 participants) leave substantial uncertainty; bounded modified Knapp–Hartung sensitivity intervals extend across large effects in both directions. Indirectness 0: HAI is a direct measure for the humoral-immunogenicity question; acute and chronic evidence is separated. No inference about prevention of clinical influenza is made. Publication bias: cannot be assessed statistically (k<10); not assumed absent and not automatically downgraded. |
| HAI titer – H3N2 Chronic | Randomized; starts high | 2 / 171 | 0.27 (-0.17 to 0.72) | Very low | RoB -1; inconsistency 0; indirectness 0; imprecision -2; publication bias: not assessable. Risk of bias −1: allocation/report-selection concerns in the contributing randomized trial(s); Woods 2009 also excludes randomized participants from its analysis. Inconsistency: no additional downgrade; sparse evidence limits interpretation of I². Imprecision −2: Two trials (171 participants) leave substantial uncertainty; bounded modified Knapp–Hartung sensitivity intervals extend across large effects in both directions. Indirectness 0: HAI is a direct measure for the humoral-immunogenicity question; acute and chronic evidence is separated. No inference about prevention of clinical influenza is made. Publication bias: cannot be assessed statistically (k<10); not assumed absent and not automatically downgraded. |
| HAI titer – Influenza B Chronic | Randomized; starts high | 2 / 171 | -0.14 (-0.64 to 0.36) | Very low | RoB -1; inconsistency 0; indirectness 0; imprecision -2; publication bias: not assessable. Risk of bias −1: allocation/report-selection concerns in the contributing randomized trial(s); Woods 2009 also excludes randomized participants from its analysis. Inconsistency: no additional downgrade; sparse evidence limits interpretation of I². Imprecision −2: Two trials (171 participants) leave substantial uncertainty; bounded modified Knapp–Hartung sensitivity intervals extend across large effects in both directions. Indirectness 0: HAI is a direct measure for the humoral-immunogenicity question; acute and chronic evidence is separated. No inference about prevention of clinical influenza is made. Publication bias: cannot be assessed statistically (k<10); not assumed absent and not automatically downgraded. |
| HAI titer – H1N1 Acute | Randomized; starts high | 1 / 57 | 0.52 (-0.51 to 1.56) | Very low | RoB -1; inconsistency 0; indirectness 0; imprecision -2; publication bias: not assessable. Risk of bias −1: allocation/report-selection concerns in the contributing randomized trial(s). Inconsistency: not assessable in a single trial; no automatic downgrade. Imprecision −2: A single small trial and a wide confidence interval permit markedly different conclusions about immunogenicity. Indirectness 0: HAI is a direct measure for the humoral-immunogenicity question; acute and chronic evidence is separated. No inference about prevention of clinical influenza is made. Publication bias: cannot be assessed statistically (k<10); not assumed absent and not automatically downgraded. |
| HAI titer – H3N2 Acute | Randomized; starts high | 1 / 57 | -0.47 (-1.38 to 0.44) | Very low | RoB -1; inconsistency 0; indirectness 0; imprecision -2; publication bias: not assessable. Risk of bias −1: allocation/report-selection concerns in the contributing randomized trial(s). Inconsistency: not assessable in a single trial; no automatic downgrade. Imprecision −2: A single small trial and a wide confidence interval permit markedly different conclusions about immunogenicity. Indirectness 0: HAI is a direct measure for the humoral-immunogenicity question; acute and chronic evidence is separated. No inference about prevention of clinical influenza is made. Publication bias: cannot be assessed statistically (k<10); not assumed absent and not automatically downgraded. |
| Seroprotection – B/Phuket/3073/2013 Acute | Randomized; Elzayat 2021; starts high | 1 / 26 | 0.31 (0.07 to 1.40) | Very low | RoB -1; inconsistency 0; indirectness 0; imprecision -2; publication bias: not assessable. Randomized evidence, downgraded once for trial limitations and twice for imprecision in a single small trial. No downgrade merely because the outcome is HAI. This estimate is not pooled with different exposure types, B antigens, or designs. |
| Seroconversion – B/Phuket/3073/2013 Acute | Randomized; Elzayat 2021; starts high | 1 / 26 | 1.25 (0.13 to 12.06) | Very low | RoB -1; inconsistency 0; indirectness 0; imprecision -2; publication bias: not assessable. Randomized evidence, downgraded once for trial limitations and twice for imprecision in a single small trial. No downgrade merely because the outcome is HAI. This estimate is not pooled with different exposure types, B antigens, or designs. |
| Seroconversion – B/Colorado/06/2017 Acute | Randomized; Elzayat 2021; starts high | 1 / 26 | 3.24 (0.17 to 61.21) | Very low | RoB -1; inconsistency 0; indirectness 0; imprecision -2; publication bias: not assessable. Randomized evidence, downgraded once for trial limitations and twice for imprecision in a single small trial. No downgrade merely because the outcome is HAI. This estimate is not pooled with different exposure types, B antigens, or designs. |
| HAI titer – B/Phuket/3073/2013 Acute | Randomized; Lee 2021; starts high | 1 / 57 | 0.21 (-0.43 to 0.85) | Very low | RoB -1; inconsistency 0; indirectness 0; imprecision -2; publication bias: not assessable. Randomized evidence, downgraded once for trial limitations and twice for imprecision in a single small trial. No downgrade merely because the outcome is HAI. This estimate is not pooled with different exposure types, B antigens, or designs. |
| HAI titer – B/Brisbane/60/2008 Acute | Randomized; Lee 2021; starts high | 1 / 57 | 0.56 (-0.04 to 1.15) | Very low | RoB -1; inconsistency 0; indirectness 0; imprecision -2; publication bias: not assessable. Randomized evidence, downgraded once for trial limitations and twice for imprecision in a single small trial. No downgrade merely because the outcome is HAI. This estimate is not pooled with different exposure types, B antigens, or designs. |
| HAI titer – H1N1 Acute | Nonrandomized; Edwards 2010; starts low | 1 / 158 | 0.24 (-0.38 to 0.85) | Very low | RoB -1; inconsistency 0; indirectness 0; imprecision -1; publication bias: not assessable. Low starting certainty for nonrandomized evidence. Serious residual confounding/selection concern beyond lack of randomization; no adjusted causal estimate. No downgrade merely because the outcome is HAI. This estimate is not pooled with different exposure types, B antigens, or designs. |
| HAI titer – H3N2 Acute | Nonrandomized; Edwards 2010; starts low | 1 / 158 | -0.09 (-0.90 to 0.71) | Very low | RoB -1; inconsistency 0; indirectness 0; imprecision -1; publication bias: not assessable. Low starting certainty for nonrandomized evidence. Serious residual confounding/selection concern beyond lack of randomization; no adjusted causal estimate. No downgrade merely because the outcome is HAI. This estimate is not pooled with different exposure types, B antigens, or designs. |
| HAI titer – Influenza B Acute | Nonrandomized; Edwards 2010; starts low | 1 / 158 | -0.12 (-0.43 to 0.19) | Very low | RoB -1; inconsistency 0; indirectness 0; imprecision -1; publication bias: not assessable. Low starting certainty for nonrandomized evidence. Serious residual confounding/selection concern beyond lack of randomization; no adjusted causal estimate. No downgrade merely because the outcome is HAI. This estimate is not pooled with different exposure types, B antigens, or designs. |
| HAI fold rise – H1N1 Acute | Randomized; Lee 2021; starts high | 1 / 57 | -0.60 (-17.95 to 16.75) | Very low | RoB -1; inconsistency 0; indirectness 0; imprecision -2; publication bias: not assessable. Randomized evidence, downgraded once for trial limitations and twice for imprecision in a single small trial. No downgrade merely because the outcome is HAI. This estimate is not pooled with different exposure types, B antigens, or designs. |
| HAI fold rise – H3N2 Acute | Randomized; Lee 2021; starts high | 1 / 57 | 0.00 (-11.67 to 11.67) | Very low | RoB -1; inconsistency 0; indirectness 0; imprecision -2; publication bias: not assessable. Randomized evidence, downgraded once for trial limitations and twice for imprecision in a single small trial. No downgrade merely because the outcome is HAI. This estimate is not pooled with different exposure types, B antigens, or designs. |
| HAI fold rise – H3N2 Chronic | Randomized; Hayney 2014; starts high | 1 / 98 | 5.16 (-3.84 to 14.16) | Very low | RoB -1; inconsistency 0; indirectness 0; imprecision -2; publication bias: not assessable. Randomized evidence, downgraded once for trial limitations and twice for imprecision in a single small trial. No downgrade merely because the outcome is HAI. This estimate is not pooled with different exposure types, B antigens, or designs. |
| HAI fold rise – Influenza B Chronic | Randomized; Hayney 2014; starts high | 1 / 98 | 1.40 (-3.57 to 6.37) | Very low | RoB -1; inconsistency 0; indirectness 0; imprecision -2; publication bias: not assessable. Randomized evidence, downgraded once for trial limitations and twice for imprecision in a single small trial. No downgrade merely because the outcome is HAI. This estimate is not pooled with different exposure types, B antigens, or designs. |
| HAI fold rise – B/Phuket/3073/2013 Acute | Randomized; Lee 2021; starts high | 1 / 57 | 5.00 (-2.76 to 12.76) | Very low | RoB -1; inconsistency 0; indirectness 0; imprecision -2; publication bias: not assessable. Risk of bias −1: incomplete reporting of missing-data handling and result selection in one small randomized trial. Inconsistency: not assessable in one trial. Indirectness: no downgrade for HAI. Imprecision −2: only 57 participants, a wide effect range and approximate reconstruction from rounded arithmetic-mean confidence limits. Publication bias is not assessable; no additional downgrade. |
| HAI fold rise – B/Brisbane/60/2008 Acute | Randomized; Lee 2021; starts high | 1 / 57 | 5.50 (0.92 to 10.08) | Very low | RoB -1; inconsistency 0; indirectness 0; imprecision -2; publication bias: not assessable. Risk of bias −1: incomplete reporting of missing-data handling and result selection in one small randomized trial. Inconsistency: not assessable in one trial. Indirectness: no downgrade for HAI. Imprecision −2: only 57 participants, a wide effect range and approximate reconstruction from rounded arithmetic-mean confidence limits. Publication bias is not assessable; no additional downgrade. The interval excludes the null using Table 3 confidence limits but includes it when the discordant SDs printed in the Results text are used; uncertainty in dispersion prevents a robust benefit inference. |
| Observed activity/fitness associations | Nonrandomized; starts low | 8 reports | Not pooled | Very low | Risk of bias −1 for important residual confounding or selection, with additional imprecision in small or incompletely reported contrasts; no upgrading. Outcome-specific Keylock judgments distinguish unadjusted seroconversion from partially adjusted continuous responses. Inconsistency cannot be quantified across incompatible outcomes; HAI does not receive an automatic indirectness downgrade; publication bias is not assessable. |

Randomized evidence starts at high certainty and nonrandomized evidence at low certainty. HAI is a direct outcome for the humoral-immunogenicity question, so indirectness is not downgraded solely because clinical influenza was not measured. Risk of bias, inconsistency, indirectness, imprecision, and publication bias are stated separately. Single-trial inconsistency and small-k publication bias are not assessable; neither is assumed absent. Illustrative absolute risks for the primary binary comparisons are provided in main Table 5. No validated patient-important HAI threshold or equivalence margin is asserted. k, number of independent comparisons; N, participants contributing to that comparison. Counts across antigens or overlapping reports must not be summed as unique participants. Lee B-antigen fold-rise profiles include uncertainty from reconstructed or conflicting dispersion, rather than using statistical significance alone to determine certainty.

**Table S7. Numerical provenance and eligibility for quantitative synthesis**

| **Report / data family** | **Source type / extraction method** | **Exact source location** | **Use in the revised analyses** |
| --- | --- | --- | --- |
| Ayling 2018 | Published correlations; no group mean reconstruction | Main Tables 3–4 (PDF pp. 23 and 26) | Observed activity correlations retained narratively; incomplete outcome-specific N prevents reliable conversion into a common quantitative effect. |
| Bachi 2013 | Published text/figures; denominator ambiguity | Table 1 and Results/Figure 1 | Group-level ELISA findings were summarized narratively. Reported n=55/55 conflicts with sex counts implying n=45/45; the analyzed denominator could not be resolved. |
| Bohn-Goldbaum 2020 | Printed Table 2; explicitly derived Ns | Table 2, PDF p. 4 | H1N1 SP/SC denominators were reconstructed as 23/21; H3N2 SP as 22/23; H3N2/B SC as 23/22. The control B SP cell, 72.7% (19), is irreconcilable with n≤23 and excluded. The logarithm base for titers is unstated. H3N2/B titers enter assumed-log10 sensitivities only. H1N1 control is printed as 1.81 ± 0.0653; an unverified decimal-place error is not corrected, and this continuous result is excluded from all effect estimates (V2_034). |
| Campbell 2010 | Narrative/figure-only outcome summary | Main antibody figures and results | No reliable arm-level means plus dispersion used. Allocation described as pseudo-randomized; no randomized primary input. |
| de Araujo 2015 | Printed event counts and denominators | Table 2, PDF p. 6 | Six-week NT denominator 15, MT 23, IT 22; unadjusted RRs calculated from printed cells. Ordered categories are within-study gradients, not a pooled dose–response curve. |
| Edwards 2006 / 2007 | Published outcome text and figures | Main antibody results/figures | Reported sex/strain patterns retained narratively. No plotted arm values estimated for the revised meta-analysis. |
| Edwards 2010 | Printed GMT and 95% CI; calculated log₂ summaries | Table 1, PDF p. 4 | Day-28 values are printed numerically, not figure-only. log₂(GMT), CI-derived SD, and standard exercise-arm combination are used. The pseudo-randomized contrast is separate from primary randomized evidence. |
| Elzayat 2021 | Printed 6-week counts; combined exercise arms | Table 3, PDF p. 7 | H1N1 SP 9/16 vs 5/10; H3N2 SP 11/16 vs 4/10; H1N1 SC 3/16 vs 1/10; H3N2 SC 1/16 vs 2/10. B/Phuket SP 2/16 vs 4/10 and SC 2/16 vs 1/10; B/Colorado SP 0/16 vs 0/10 and SC 2/16 vs 0/10. Distinct B antigens remain separate. All counts are taken from the printed 6-week columns after combining the two exercise arms. |
| Felismino 2021 | Published immunoglobulin findings | Results/Figure 3, PDF p. 8 | Observed-exposure narrative; no figure-derived means used. Cohort overlap with Monteiro is retained in the report linkage. |
| Hallam 2022 | Exploratory figure digitization; excluded | Supplementary figures S4a, S4b, S5a, S5b, S5e, and S5f | Earlier exploratory readings concerned ELISA antibody fold changes at two and four weeks in the monovalent H1N1 and seasonal influenza experiments. Overlapping individual markers and repeated observations prevent a reliable independent arm-level reconstruction. No figure-derived values enter any revised primary or sensitivity estimate. These optical-density outcomes remain narrative and are not HAI titers or HAI fold rise. |
| Hayney 2014 | Printed Table 2; ambiguous cells excluded | Table 2, PDF p. 5 | H1N1 SP and all three SC count/N pairs are used directly. H3N2/B SP cells are inconsistent; H3N2 hypothetical alternatives appear only in sensitivity analyses. H1N1 raw fold-rise means are excluded as incompatible with the printed ≥4-fold responder counts. H3N2/B mean fold rises and SDs are reported as single-trial supplementary estimates; unclear GMT dispersion is not assumed to be SD. |
| Keylock 2007 | Printed seroconverter counts | Seroconversion discussion, PDF p. 7; covariate analyses, PDF pp. 3 and 6 | H1N1 8/13 versus 3/13 and influenza B 7/13 versus 2/13 yield unadjusted event-count RRs. These are distinct from the article’s covariate-adjusted continuous-response analyses. High-risk confounding judgments for the binary estimates are not replaced by the more favorable continuous-outcome judgments. |
| Kohut 2002 | Published ELISA results and symptom counts | Results/Figures 1–2, PDF pp. 4–5 | ELISA results are narrative. Influenza-like illness 3/25 vs 1/15 is moderately active vs sedentary; active participants had 0/16. The illness observation is not pooled as a humoral outcome. |
| Kohut 2004 | Printed log₂ mean ± SE | Table 2, PDF p. 5 | Post-training year-2 vaccination, 4-week follow-up; n=14/13. Variance uses SE_exercise²+SE_control². Young comparison participants and first-year prevaccination observations are not counted as randomized training arms. |
| Lee 2021 | Printed GMT/CI and arithmetic fold-rise CI | Tables 3–4, PDF pp. 5–6 | Titer: log₂(GMT) and confidence-limit-derived SDs; two n=19 exercise arms combined against n=19 control. Fold rise: published combined-exercise means and approximate SEs from arithmetic confidence limits, including both B/Phuket and B/Brisbane. Table 3 B/Brisbane control mean 2.1 (95% CI 1.5–2.6) differs from Results text 2.0 ± 11.1; the text also gives exercise 7.6 ± 12.9. V2_043/044 retain table-based B-antigen estimates; V2_045 is a text-SD sensitivity. Antigens are not pooled together. Vaccine dose was 0.25 mL. |
| Long 2012 | Narrative/figure and responder results | Results/Figure 3, PDF p. 5 | No complete compatible arm-level HAI mean/dispersion input was derived; age-stratum findings are not conflated with an age interaction. |
| Monteiro 2020 | Published text/HAI figure | Results/Figure 2, PDF p. 8 | Only the placebo-arm training vs non-practicing exposure contrast is eligible; supplementation effects are not attributed to exercise. No plotted mean/SD is digitized into the primary analysis. |
| Ranadive 2014 | Published protective-response proportions and sample sizes | Table 2, PDF p. 5; Table 1, PDF p. 3 | H1N1 11/27 vs 14/28; H3N2 18/27 vs 20/28; B 1/27 vs 3/28. Counts are reconstructed from the printed distribution of seroprotected participants, not from antibody bar heights. |
| Sadarangani 2022 | Published adjusted fold-rise ratios | Main Table 2, PDF p. 5 | RAPA-category adjusted ratios and their 95% CIs are retained as within-study observational estimates. They are not mixed with arm-level randomized titer MDs. |
| Woods 2009 | Printed log₂ means/SEM and numerical figure labels | Table 3, PDF p. 5; Figure 2, PDF p. 6 | Primary titers use 3-week means/SEM (74/70). The printed 24-week protection counts are H1N1 32/74 vs 15/70, H3N2 41/74 vs 27/70, B 34/74 vs 25/70; these are printed labels, not digitized coordinates, and are outside the peak window. |
| Yang 2007 | Published longitudinal/composite findings | Results/Figure 2 and model, PDF pp. 7–8 | Nonrandomized/mixed-control comparison; no compatible randomized strain-specific estimate. No individual-level author data or figure-based HAI means are used. |
| Investigator contact / unpublished data | No contact; no unpublished data used | Published articles and their supplements only | Original investigators were not contacted. Given the age of several foundational trials and uncertainty about archived-data availability, numerical retrieval was limited to published articles and supplements. Missing or inconsistent values therefore could not be clarified by investigators. |

Printed tables, text, and numerical figure labels are distinguished from readings of graphical coordinates. The machine-readable source data preserve the published inputs, reconstruction formulas, source locations, and analysis status. Numerical ambiguities are described explicitly rather than resolved by selecting an assumed value. Source-dispersion alternatives are not independent observations.

**Figure S1. Individual HAI fold-rise estimates, separated by exposure and antigen.**

Individual mean differences (MDs) in raw HAI fold rise from Lee (2021; acute exercise) and Hayney (2014; chronic training). No pooled diamond or acute-plus-chronic subtotal is shown. Both Lee B antigens are retained separately. Its estimates use approximate SEs reconstructed from the arithmetic-mean confidence limits in Table 3. The B/Brisbane interval excludes zero in this table-based calculation but includes zero when the discordant text-reported SDs are used (Table S4); the plotted interval therefore does not establish a robust benefit. Hayney H1N1 is excluded because its printed means cannot be reconciled as raw fold ratios with the ≥4-fold responder counts. Each row is a distinct antigen-specific comparison, not an independent participant cohort. CI, confidence interval; HAI, hemagglutination inhibition.

**Figure S2. Within-study observational exposure gradients.**

The top plot displays unadjusted seroprotection RRs at six weeks by training lifestyle in de Araujo (2015), and the lower plot displays multivariable-adjusted HAI fold-rise ratios by RAPA category in Sadarangani (2022). The reported H3N2 linear-trend p value in Sadarangani was 0.015. Both reports were assessed with ROBINS-E and were at high risk of bias. The curves connect observed category estimates for readability and are not fitted dose–response functions; the plotted reference category has no independently estimated confidence interval. CI, confidence interval; RAPA, Rapid Assessment of Physical Activity; RR, risk ratio.
